# A New Upper Jurassic Ophthalmosaurid Ichthyosaur from the Slottsmøya Member, Agardhfjellet Formation of Central Spitsbergen

**DOI:** 10.1371/journal.pone.0103152

**Published:** 2014-08-01

**Authors:** Aubrey Jane Roberts, Patrick Scott Druckenmiller, Glenn-Peter Sætre, Jørn Harald Hurum

**Affiliations:** 1 The Natural History Museum, University of Oslo, Oslo, Norway; 2 University of Alaska Museum, University of Alaska Fairbanks, Fairbanks, Alaska, United States of America; 3 Department of Geology and Geophysics, University of Alaska Fairbanks, Fairbanks, Alaska, United States of America; 4 Centre for Ecological and Evolutionary Synthesis, Department of Biosciences, University of Oslo, Oslo, Norway; Raymond M. Alf Museum of Paleontology, United States of America

## Abstract

Abundant new ichthyosaur material has recently been documented in the Slottsmøya Member of the Agardhfjellet Formation from the Svalbard archipelago of Norway. Here we describe a partial skeleton of a new taxon, *Janusaurus lundi*, that includes much of the skull and representative portions of the postcranium. The new taxon is diagnosed by a suite of cranial character states including a very gracile stapedial shaft, the presence of a dorsal process on the prearticular and autapomorphic postcranial features such as the presence of an interclavicular trough and a conspicuous anterodorsal process of the ilium. The peculiar morphology of the ilia indicates a previously unrecognized degree of morphological variation in the pelvic girdle of ophthalmosaurids. We also present a large species level phylogenetic analysis of ophthalmosaurids including new and undescribed ichthyosaur material from the Upper Jurassic of Svalbard. Our results recover all Svalbard taxa in a single unresolved polytomy nested within Ophthalmosaurinae, which considerably increases the taxonomic composition of this clade. The paleobiogeographical implications of this result suggest the presence of a single clade of Boreal ophthalmosaurid ichthyosaurs that existed during the latest Jurassic, a pattern also reflected in the high degree of endemicity among some Boreal invertebrates, particularly ammonoids. Recent and ongoing descriptions of marine reptiles from the Slottsmøya Member Lagerstätte provide important new data to test hypotheses of marine amniote faunal turnover at the Jurassic-Cretaceous boundary.

## Introduction

**Figure 1:**
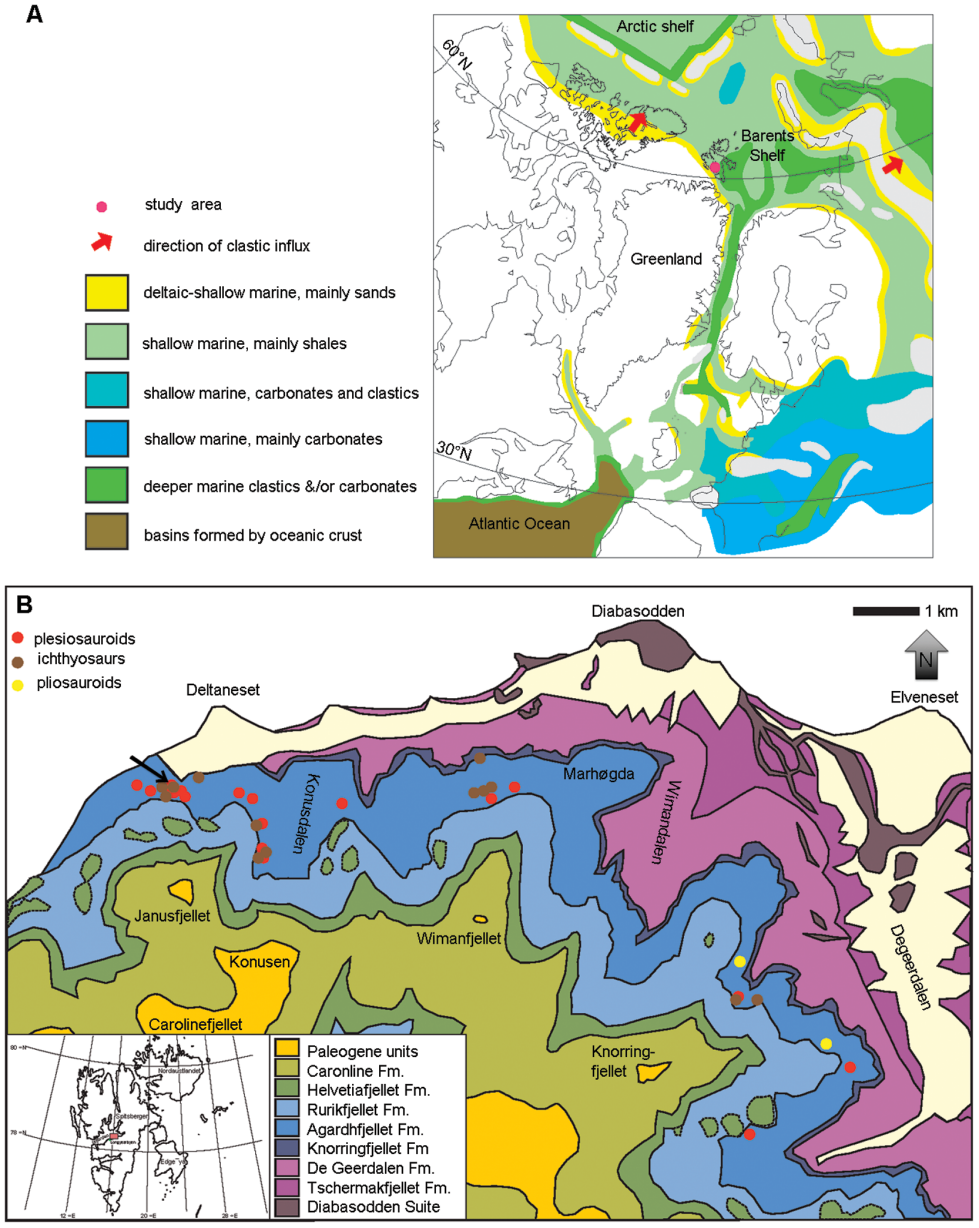
Maps of the study area in Svalbard, Norway. A: Palaeogeographic map of the North Atlantic region in the Late Jurassic, modified from Torsvik et al. [2]. The red dot locates the study area; B: Geological map of the study area including the Agardhfjellet Formation and dig sites. The black arrow points to the site where specimen PMO 222.654 was excavated. Modified from Hurum et al. [1] and Dallmann et al. [14].

From 2004–2012, eight seasons of fieldwork in the Late Jurassic to earliest Cretaceous Slottsmøya Member of the Agardhfjellet Formation in the central Spitsbergen Sassenfjord area, have yielded numerous skeletal remains of marine amniotes (Figure 1) [1], [2]. Through a number of papers, these Arctic localities have been documented [3]–[14], which built the framework for what is now known as the Slottsmøya Member Lagerstätte (SML), which resulted in the description of two new ophthalmosaurid ichthyosaur taxa; *Cryopterygius kristiansenae* and *Palvennia hoybergeti* [15], as well as five new plesiosaurians [16]–[19]. Here, we present a description of a third new ophthalmosaurid taxon from the Slottsmøya Member, which includes most of the skull, girdle elements and fore- and hind limbs.

The specimen, PMO 222.654, is significant in that it represents one of the stratigraphically oldest specimens excavated from the Slottsmøya Member and provides important new morphological data for comparisons with age pene-contemporaneous material from the Kimmeridge Clay Formation of the U.K. and elsewhere. An ever-expanding body of data on ichthyosaurs from the SML demonstrates previously unrecognized diversity among Late Jurassic taxa, similar to recent work recognizing high diversity among Early Cretaceous ichthyosaurs [20]. Collectively, the SML assemblage is also significant in being one of only two major marine amniote sites that span the Jurassic-Cretaceous boundary (the other being the Vaca Muerta Formation of Argentina [21], [22]), thereby contributing to ongoing discussions regarding potential marine reptile turnover at the Jurassic-Cretaceous boundary [23].

### Geological Setting

The Slottsmøya Member is the uppermost of four members in the Agardhfjellet Formation [24]. It is overlain by the Myklegardfjellet Bed, which is the base of the Rurikfjellet Formation [24]. These two formations form the Janusfjellet Subgroup, which is part of the Adventdalen group first described by Parker [25]. The Janusfjellet Subgroup is Late Jurassic to Early Cretaceous in age and is interpreted to be a marine shelf to prodeltaic succession dominated by shale, with subordinate siltstone and sandstone [24], [25]. The Slottsmøya Member consists of 55-70 meters of dark-grey to black silty mudstone, often weathered into paper shale. There are discontinuous silty beds, with occurrences of siderite and dolomite interbeds and yellow-to-red sideritic concretions [24]. The Slottsmøya Member records a transgressive and subsequent regressive period, with varying degrees of dysaerobic sea bottom conditions [3], [26]. There was a low sedimentation rate and relatively high organic productivity in the upper water column, leading to significant accumulations of organic matter in the bottom sediments, reaching 5% in some layers [4], [27].

**Figure 2:**
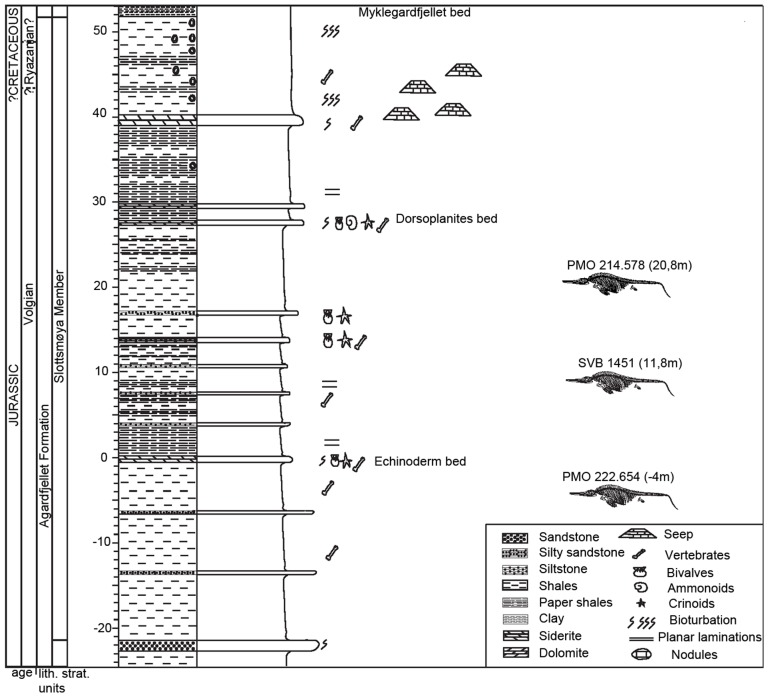
The stratigraphic position the described Spitsbergen ophthalmosaurids. *Janusaurus lundi* (PMO 222.654) in reference to *Palvennia hoybergeti* (SVB 1451) and *Cryopterygius kristiansenae* (PMO 214. 578). Adapted from Hurum et al. 2012.

The Slottsmøya Member has been dated biostratigraphically from the Upper Volgian to Upper Ryazanian, which corresponds to about 12 million years of deposition [4], [6]. The member has been divided into three units following Collignon & Hammer [3]. The lowest unit extends from the base of the member (−22 m) to a yellow echinoderm marker bed. The middle unit, for which a high degree of stratigraphic resolution has been established, extends from the yellow echinoderm bed (0) to the *Dorsoplanites* bed (27 m), and represents 5 Ma of deposition. The upper unit ranges from the *Dorsoplanites* bed (27 m) to the *Myklegardfjellet* Beds, which is a condensed section and represents approximately 7 Ma of deposition. Evidence of carbonate seeps have also been described from the upper unit [6], [7]. The specimen described in this paper (PMO 222.654) is from the lower section and is estimated to be approximately 3 Ma older than *Cryopterygius kristiansenae* and 2 Ma older than *Palvennia hoybergeti* (Figure 2).

## Materials and Methods

The holotype and only known specimen of *Janusaurus lundi*, PMO 222.654, was excavated in 2010 from the Slottsmøya Member at Janusfjellet (Janus Mountain) in central Spitsbergen. The specimen was prepared and is housed in the Geological Museum, at the University of Oslo, Norway. All necessary permits were obtained for the described study, which complied with all relevant regulations. The following permit was given by the Governor of Svalbard to excavate the specimen in 2010: 2006/00528-24.

### Institutional Abbreviations

CAMSM, Sedgwick Museum of Earth Science, Cambridge University, Cambridge, UK;

GLAHM, The Hunterian Museum, University of Glasgow, Glasgow, UK;

NHMUK, Natural History Museum, London, UK;

OUM, Oxford University Museum of Natural History, Oxford, UK;

PMO, Paleontological Museum Oslo, University of Oslo, Oslo, Norway;

SVB, Svalbard Museum, Longyearbyen, Norway.

### Phylogenetic analysis

A new phylogenetic analysis of Ophthalmosauridae was performed based on 22 taxa and 56 characters, using a modified version of the matrix in Fischer et al. [23] (see Text S1 and Table S1 for character list and data matrix). Several new taxa were added to the analysis, including PMO 222.654 (holotype of *Janusaurus lundi*), *Cryopterygius kristiansenae*, *Palvennia hoybergeti*, *Undorosaurus gorodischensis*., *Malawania anachronus*, *Leninia stellans* and an undescribed specimen from the Agardhfjellet Formation of Svalbard, PMO 222.667 [15], [28]–[30]. The new matrix includes the majority of undisputed ophthalmosaurids except *Ophthalmosaurus natans* (a putative synonym of *Ophthalmosaurus icenicus*) and *Nannopterygius enthekiodon*, which is poorly described and currently unavailable for study [31].

We further modified the matrix by the addition of five new characters (11, 12, 16, 25 & 52), relating to the nature of the anterior and posterior extension of the jugal, the morphology of the postfrontal-postorbital contact, the thickness of the stapedial shaft, and the ventral process on femur (see Text S2).

Finally, we critically reevaluated the existing scores based on personal observation and the recent literature for *Ophthalmosaurus icenicus*, *Stenopterygius quadriscissus*, *Mollesaurus periallus*, *Caypullisaurus bonapartei*, *Brachypterygius extremus*, and *Arthropterygius chrisorum* [32]–[34] (see Text S2). Characters were not weighted or ordered, and the search was an implicit enumeration. The program TNT [35] was used to analyze the character matrix and calculate Bremer Support and bootstrap values A bootstrap analysis was run in TNT with 1000 replicates, using the TBR algorithm.

### Nomenclatural Acts

The electronic edition of this article conforms to the requirements of the amended International Code of Zoological Nomenclature, and hence the new names contained herein are available under that Code from the electronic edition of this article. This published work and the nomenclatural acts it contains have been registered in ZooBank, the online registration system for the ICZN. The ZooBank LSIDs (Life Science Identifiers) can be resolved and the associated information viewed through any standard web browser by appending the LSID to the prefix “http://zoobank.org/”. The LSID for this publication is: urn:lsid:zoobank.org:pub:FF4834F1-AEED-4B08-8E74-7125801C1B3E

The electronic edition of this work was published in a journal with an ISSN, and has been archived and is available from the following digital repositories: PubMed Central, LOCKSS and CRIStin (University of Oslo Library).

## Results

### Systematic Paleontology

ICHTHYOSAURIA de Blainville 1835

Neoichthyosauria Sander 2000

Thunnosauria Motani 1999

Ophthalmosauridae Baur 1887

Janusaurus gen. nov.

urn:lsid:zoobank.org:act:4D77CFCF-22A0-4899-A619-1E93D1ADF3C5

Janusaurus lundi sp. nov.

urn:lsid:zoobank.org:act:71E65B35-7215-44AA-BCE7-E9A3B265E04F

(Figures. 3-14)

**Figure 3:**
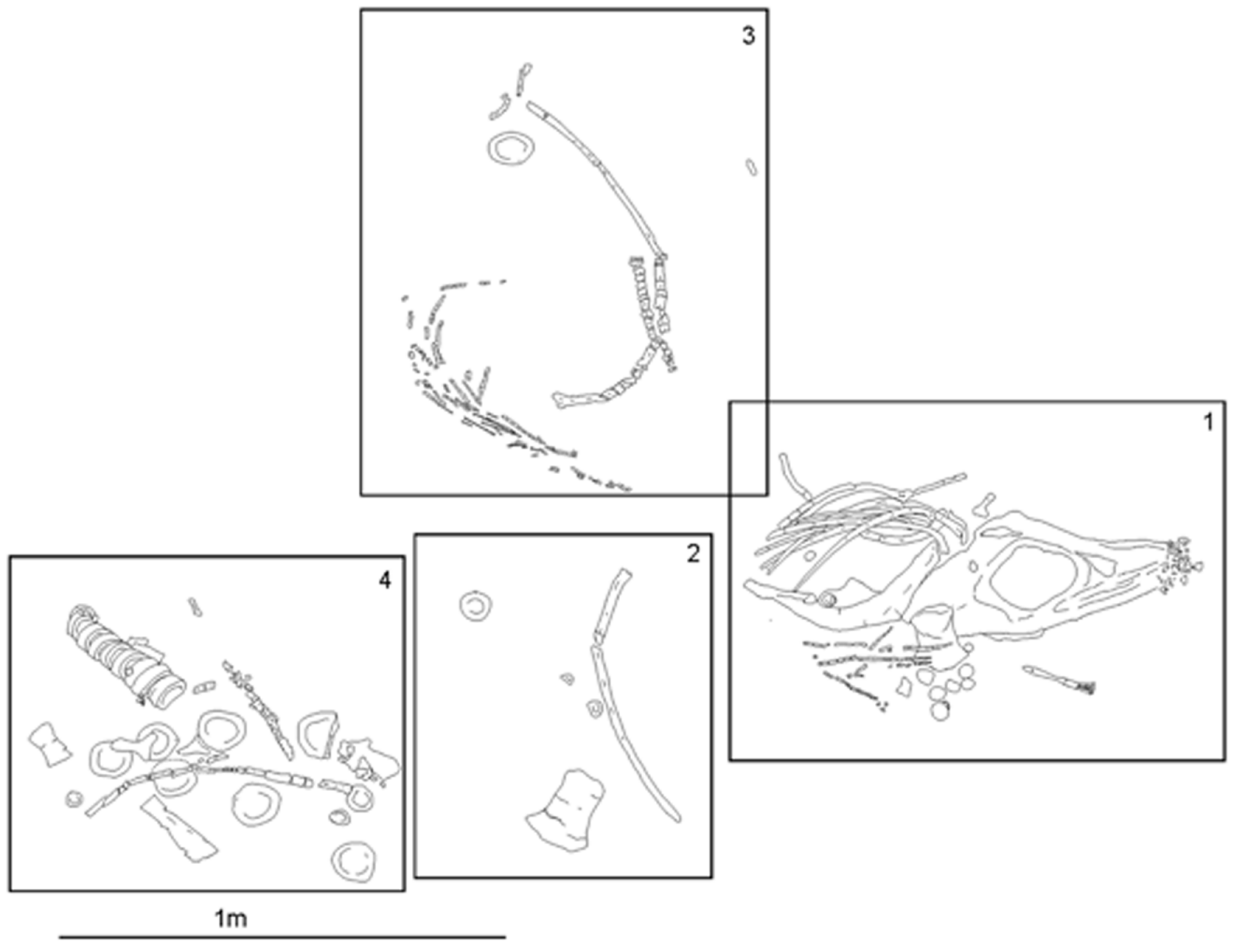
Skeleton of PMO 222.654. The specimen is presented showing the better preserved (stratigraphical-down) side after it was prepared. Dashed lines indicate boundaries of each of the four field jackets made at the time of collection. (Adapted from Novis [36]). Scale = 1 m.

**Figure 4:**
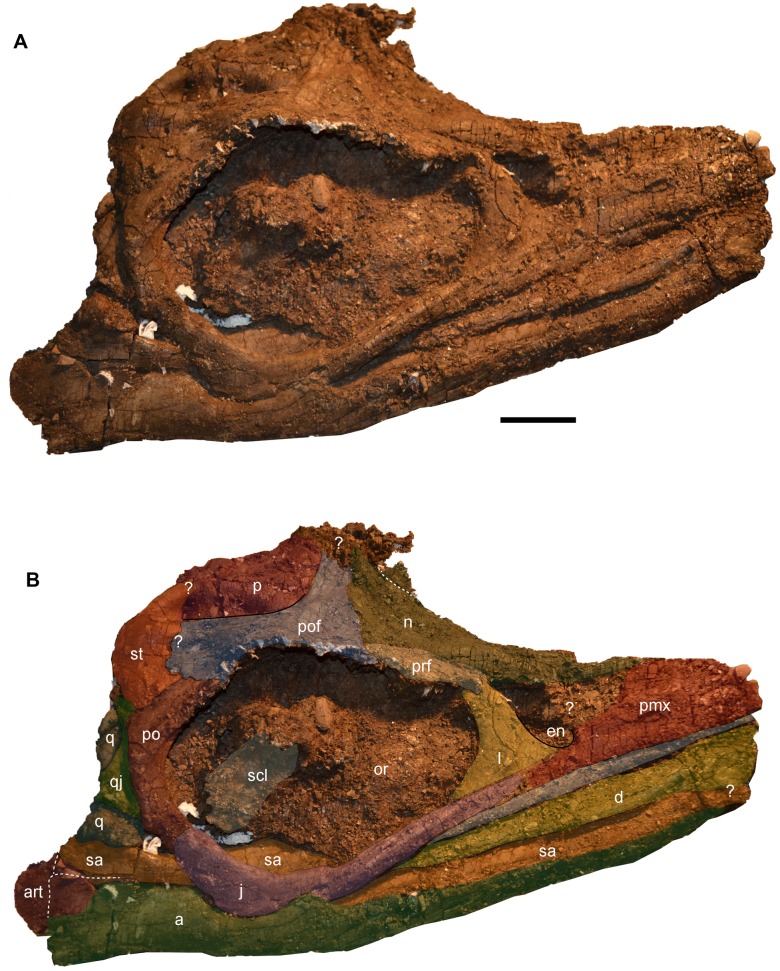
The skull of *Janusaurus lundi* (PMO 222.654). A: Photo in right lateral view of the skull. B: Right lateral view with interpretation of the individual elements. Abbreviations: a, angular; art, articular; d, dentary; en, external naris; j, jugal; l, lacrimal; mx, maxilla; n, nasal; or, orbit; p, parietal; pmx, premaxilla; po, postorbital; pof, postfrontal; prf, prefrontal; q, quadrate; qj, quadratojugal; sa, surangular; st, supratemporal. Scale = 5 cm.

**Figure 5:**
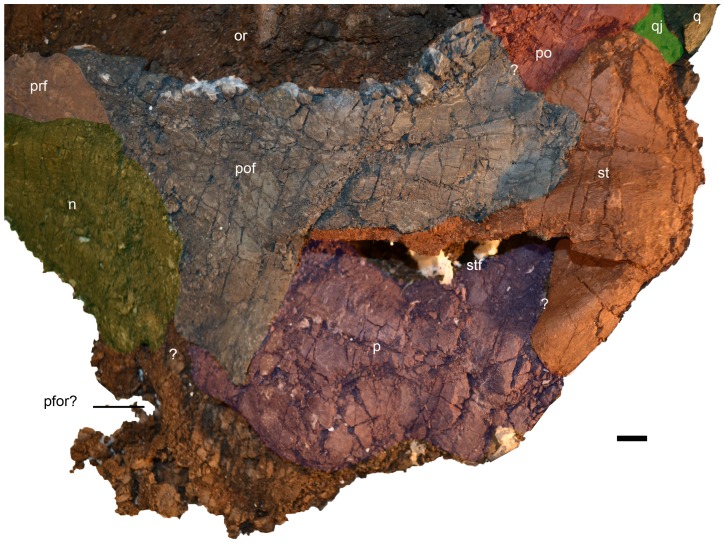
Oblique dorsal view of the skull of *Janusaurus lundi* (PMO 222.654). The dotted lines mark eroded or equivocal sutures. Abbreviations: p, parietal; pfor, pineal foramen; prf, prefrontal; pof, postfrontal; po, postorbital; q, quadrate; qj, quadratojugal; st, supratemporal; stf, supratemporal fenestra. Scale = 1 cm.

**Figure 6:**
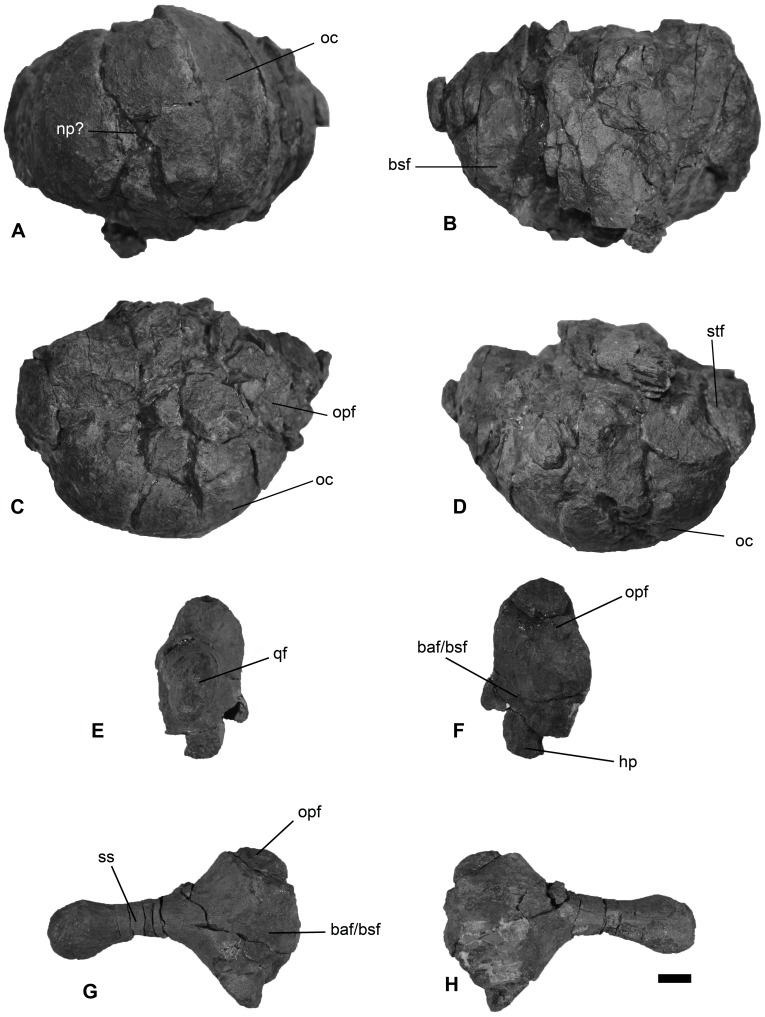
The basioccipital and right stapes from *Janusaurus lundi* (PMO 222.654). A: posterior view of the basioccipital; B: anterior view of the basioccipital; C: dorsal view of the basioccipital; D: ventral view of the basioccipital; E: lateral view of the right stapes, F: medial view of the medial head of the right stapes; G: anterior view of the right stapes; H: posterior view of the right stapes. Abbreviations: baf/bsf, basioccipital and basisphenoid facet;bsf, basiospenoid facet; hp, hyoid facet; oc, occipital condyle; opf, opisthotic facet; qf, quadrate facet; stf, stapes facet. Scale = 1 cm.

**Figure 7:**
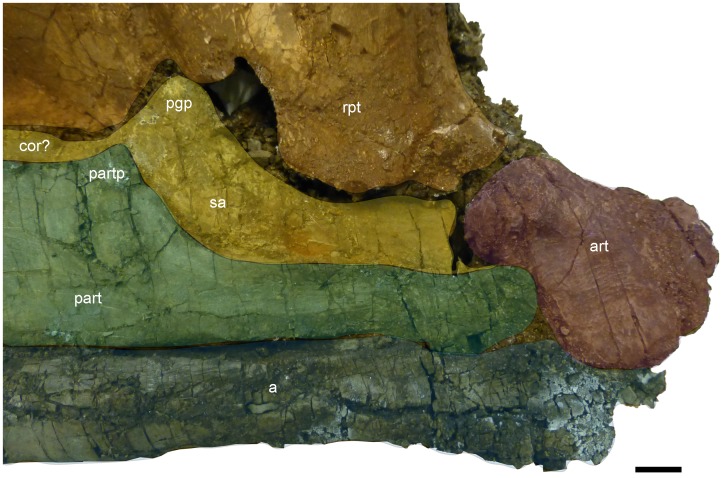
Medial view of the preserved right mandible of *Janusaurus lundi* (PMO 222.654). Medial view of the posterior portion of the right mandible and the posterior portion of the palate. Abbreviations: a, angular; art, articular; cor, coronid process; part, prearticular; partp, prearticular dorsal process; pgp, preglenoid process; pt, right pterygoid; sa, surangular. Scale = 1 cm.

**Figure 8:**
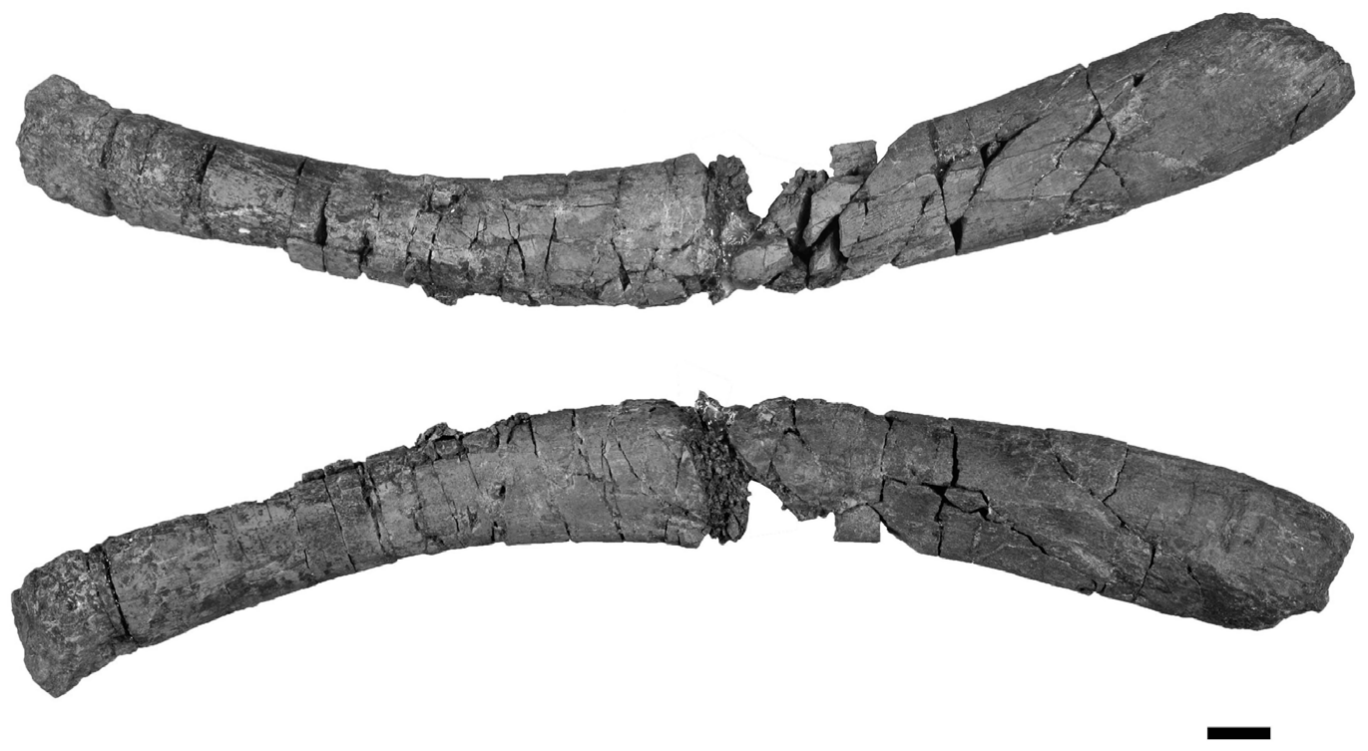
The right hyoid from *Janusaurus lundi* (PMO 222.654). Posterior is to the left, anterior to the right. Ventral view is situated at the top, dorsal view below. Scale = 1 cm.

**Figure 9:**
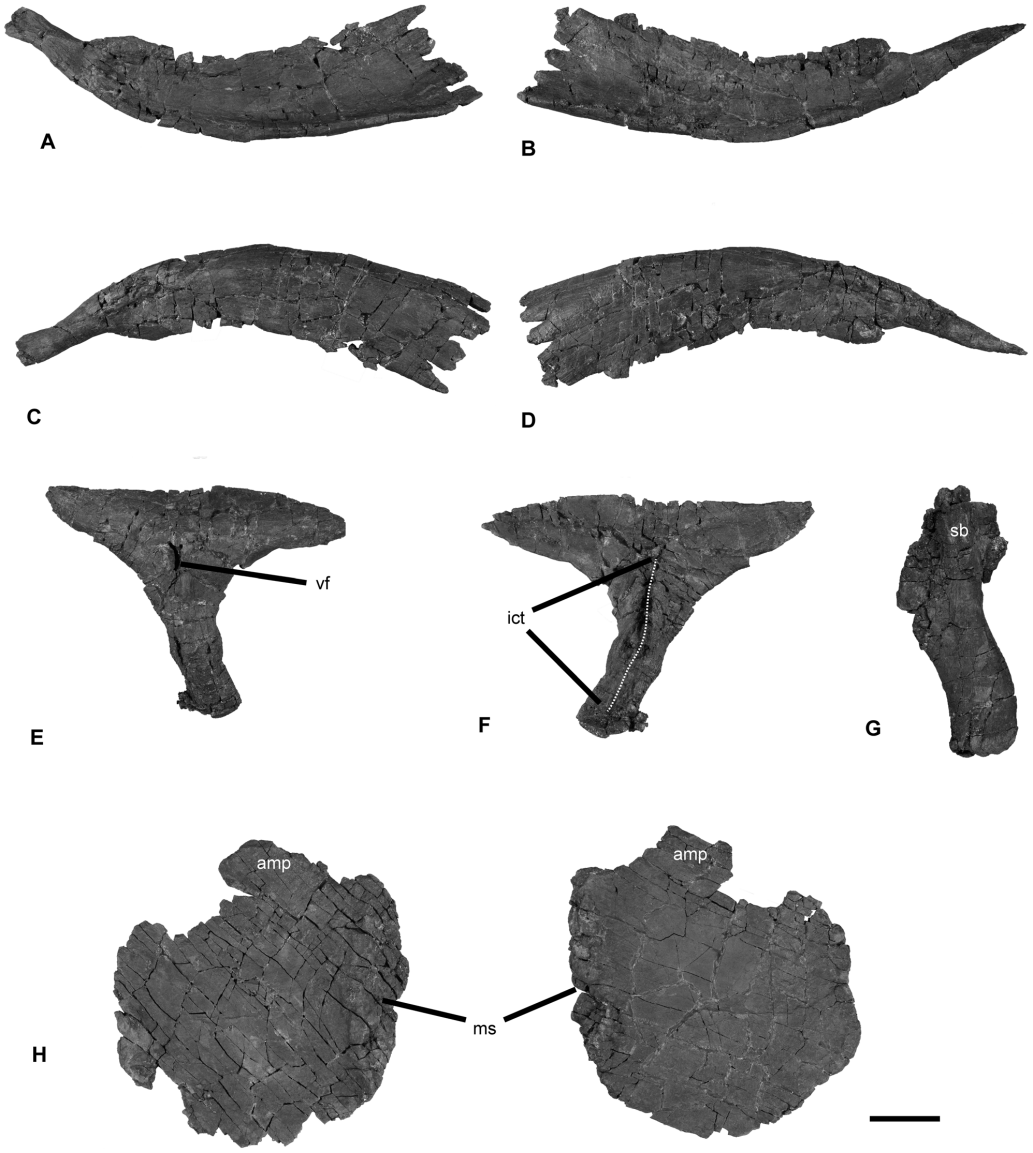
Pectoral girdle of *Janusaurus lundi* (PMO 222.654). A: posterior view of the left clavicle; B: posterior view of the right clavicle; C: anterior view of the left clavicle; D: anterior view of the right clavicle; E: ventral view of the interclavicle; F: dorsal view of the interclavicle; G: dorsal? view of the right scapula; H: dorsal view of the left coracoid; I: dorsal view of the right coracoid. Abbreviations: amp, anteromedial process; ict, interclavicular trough; ms, medial symphysis; sb, scapular blade; vf, ventral foramen. Scale = 5 cm.

**Figure 10:**
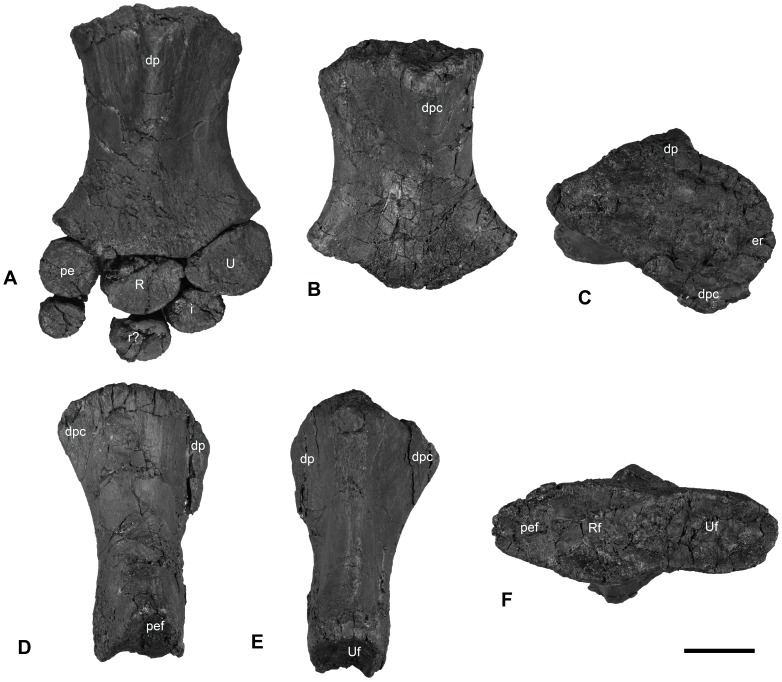
Left humerus of PMO 222.654. A: dorsal view with articulating elements; B: ventral view; C: proximal view; D: anterior view; E: posterior view; F: distal view. Abbreviations: er, elevated rim; dpc, deltopectoral crest; dp, dorsal process; pe, preaxial accessory element; pef, preaxial accessory element facet; i, intermedium; r, radiale; R, radius; Rf, radial facet; U, ulna; Uf, ulnar facet. Scale = 5 cm.

**Figure 11:**
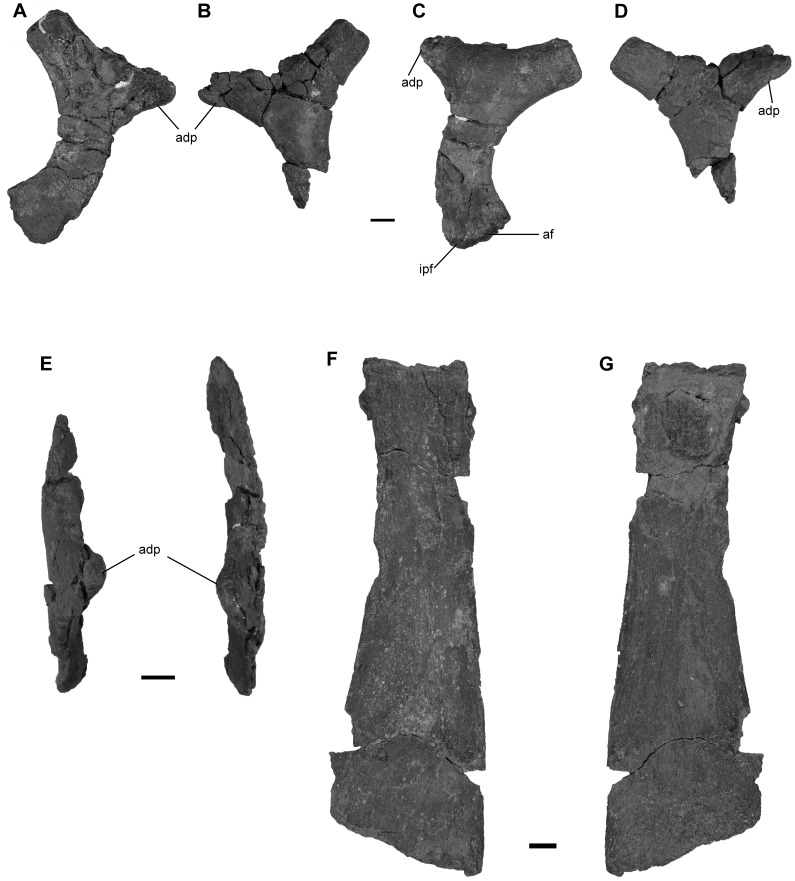
Ilia and left ischiopubis of *Janusaurus lundi* (PMO 222.654). A: lateral view of the right ilium; B: lateral view of the left ilium; C: medial view of the right ilium; D: medial view of the left ilium; E: dorsal view ilia; F: medial side of the left ischiopubis; G: lateral side of the left ischiopubis. Abbreviations: adp, anterodorsal process; af, acetabular facet; ipf, ischiopubic facet. Scale = 1 cm.

**Figure 12:**
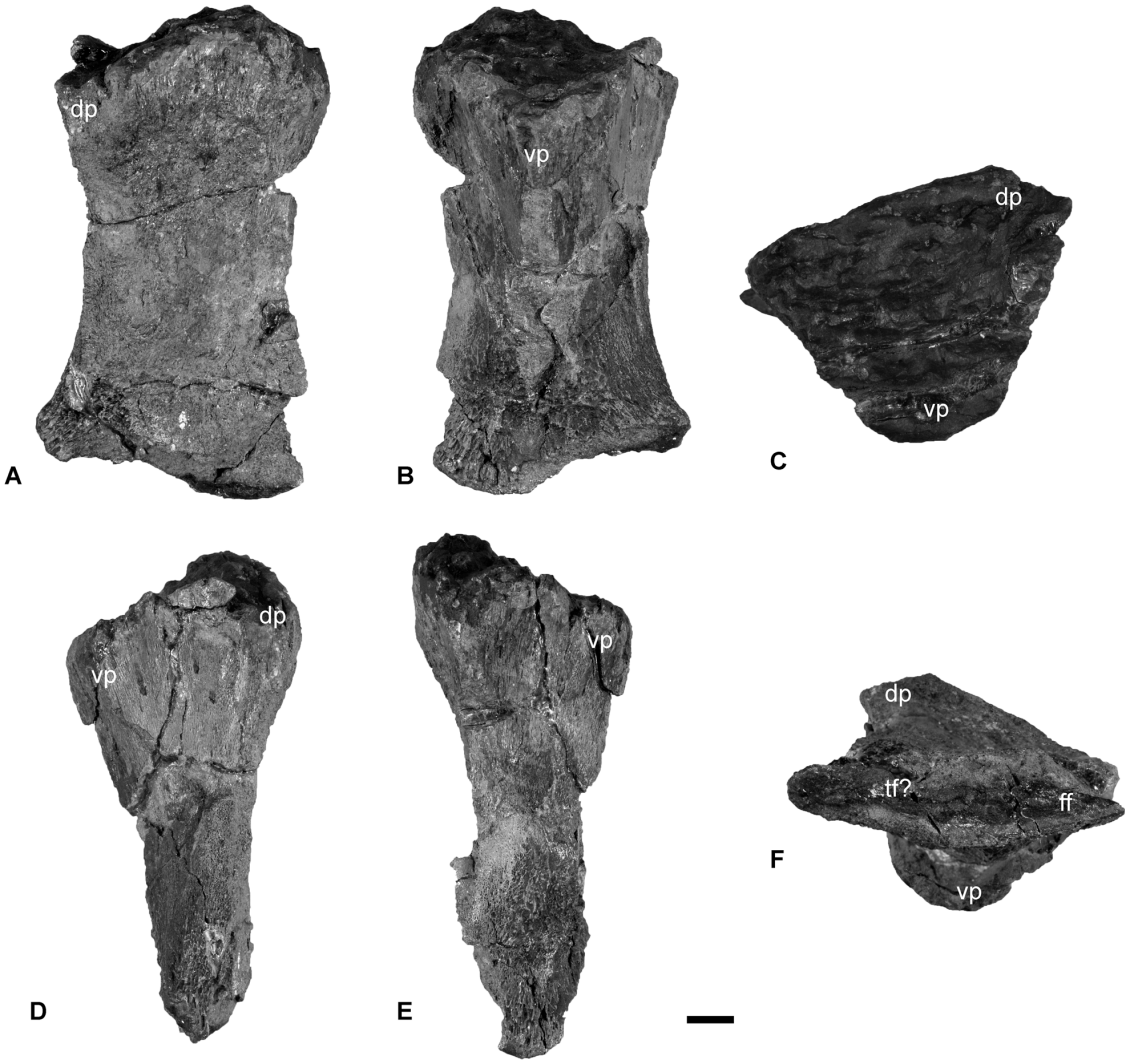
The left femur of *Janusaurus lundi* (PMO 222.654). A: dorsal view; B: ventral view; C: proximal view; D: anterior view; E: posterior view; F: distal view. Abbreviations: dp, dorsal process; ff, fibular facet; tf, tibial facet; vp, ventral process. Scale = 1 cm.

**Figure 13:**
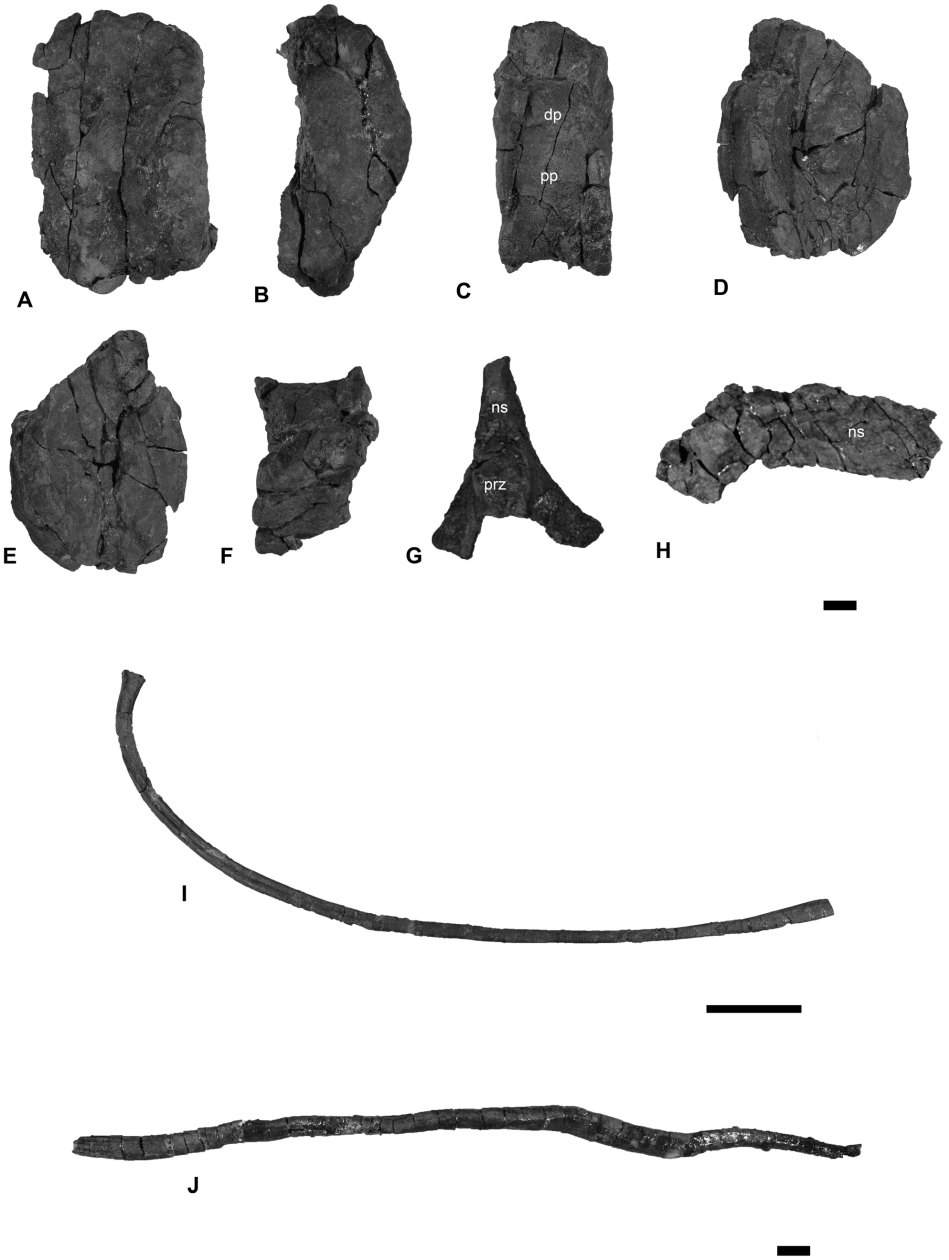
The axial skeleton of *Janusaurus lundi* (PMO 222.654). Including the partial atlas-axis complex, complete trunk vertebra, neural arches, dorsal rib and gastralia from PMO 222.654. A: lateral? view of atlas-axis complex; B: anterior view of atlas-axis complex; C: lateral view of trunk vertebrae; D: anterior view of trunk vertebrae; E: posterior view of trunk vertebrae; F: dorsal view of trunk vertebrae; G: neural arch from the sacral region in anterior view; H: neural arch from the trunk region in lateral view; I: nearly complete dorsal rib; J: complete gastralium. Abbreviations: prz, prezygapophysis; dp, diapophysis; ns, neural spine; pp, parapophysis. A-H Scale = 1 cm, I Scale = 10 cm, J Scale = 1 cm.

**Figure 14:**
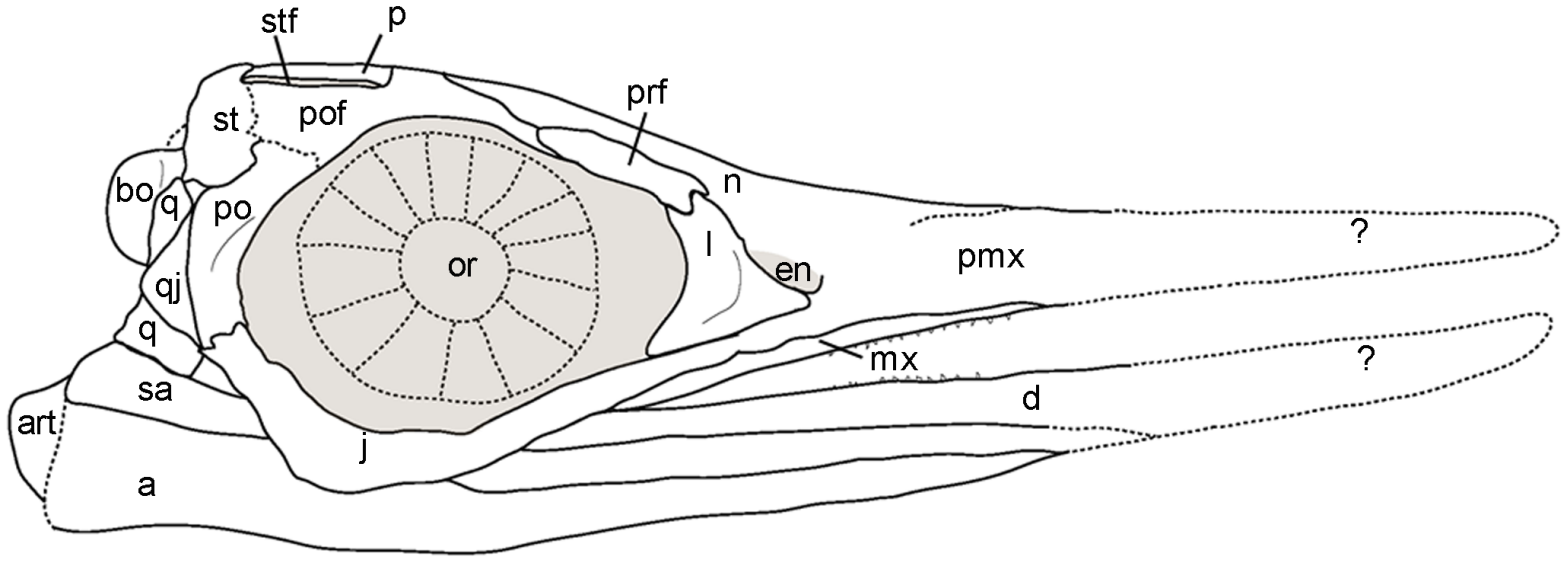
Skull reconstruction of *Janusaurus lundi* in right lateral view. Abbreviations: a, angular; art, articular; bo, basioccipital; d, dentary; en, external naris; j, jugal; l, lacrimal; mx, maxilla; n, nasal; or, orbit; p, parietal; pmx, premaxilla; po, postorbital; pof, postfrontal; prf, prefrontal; q, quadrate; qj, quadratojugal; sa, surangular; st, supratemporal; stf, supratemporal fenestra.

**Holotype and only specimen:** PMO 222.654, an incomplete skeleton consisting of a partial skull, representative cervical, dorsal and caudal vertebrae, a nearly complete pectoral girdle and left forefin, the right humerus, a partial pelvic girdle and both femora.

**Etymology**: Genus name after the mountain Janusfjellet, on which the specimen was found. Species name in honor of Bjørn Lund, technician on the excavations in 2006-2012.

**Holotype locality**: North side of Janusfjellet, ∼13 km northeast of Longyearbyen, Spitsbergen, Svalbard, Norway. UTM: N78 20.264 E15 50.044

**Holotype horizon and stage**: Slottsmøya Member, Agardhfjellet Formation, early Middle Volgian, Upper Jurassic; 31 m below the *Dorsoplanites* Bed, 4 m below the echinoderm bed [1].

### Differential diagnosis

A moderately sized ophthalmosaurid (estimated body length of 3-4 meters) possessing the following autapomorphies (marked with *) and unique character combinations: maxilla with extensive lateral exposure (short in *Ophthalmosaurus* and *Aegirosaurus*); lacrimal contributes to the posterior margin of the external naris (excluded in *Cryopterygius* and *Athabascasaurus*); posterodorsal process of jugal forming half of the posterior margin of the orbit (does not form any of the margin in *Cryopterygius*); narrow postorbital bar (broad in *Cryopterygius*, *Athabascasaurus* and *Brachypterygius*); absence of a squamosal (present in *Athabascasaurus* and *Aegirosaurus*); extremely gracile and constricted stapedial shaft*; reduced ophisthotic facet on the basioccipital (large in *Palvennia*); presence of an angular-articular contact*; extremely gracile dentition (more robust in *Cryopterygius* and *Brachypterygius*); interclavicle with an interclavicular trough and ventral foramen*; proximodistal length of scapula very reduced in comparison to coracoid length*; humerus with three distal facets (two in *Nannopterygius* and C*ryopterygius*); ulna is the largest element of zeugopodium (radius is larger in *Cryopterygius*); anterodorsal process of the ilium*; ischiopubis completely fused and lacking an obturator foramen (unfused distally in *Cryopterygius* and *Undorosaurus* and oburator foramen present in *Ophthalmosaurus*); femur with two distal facets (three in *Platypterygius americanus* and *Platypterygius australis*).

### Description

The estimated length of the animal is 3–4 m, based on comparisons of skull and rib size in other more complete ophthalmosaurids. The preserved portion of the skull is similar in size and relative proportions to that of *Palvennia hoybergeti*, which is estimated to be a small to moderately sized (3–4 m) species [15].

**Taphonomy**: The holotype specimen of *Janusaurus lundi* PMO 222.654, is an incomplete, partially articulated skeleton, collected in four jackets (Figure 3) [36]. The skull, pectoral girdle, left forelimb, and cervical vertebrae are closely associated. The right humerus was disarticulated from the rest of the pectoral girdle. Several ribs and gastralia were clustered together posterior to the skull, as was the remaining material, including the presacral and caudal vertebrae, left pelvic girdle and two femora. Several vertebrae and the right ilium were collected as surface material in the field. The individual appears to have come to rest on the sea floor on its right ventrolateral side, which is also better preserved. Because the specimen was collected in permafrost, the individual bones are broken into millimeter-sized fragments due to congelifraction, which is typical for all the marine reptile specimens from the Slottsmøya Member [15].

An isolated tooth was associated with the skeleton of PMO 222.654 (Figure S2 K). The tooth was discovered during preparation and was located in the vicinity of the disarticulated right humerus. The tooth is incomplete and lacks the apex of the crown and measures 3.5 mm in total length. It is very gracile and needle-like, similar in overall morphology to teeth associated with the plesiosaur *Spitrasaurus larseni* (SVB 1450) [16]. For this reason, we attribute the tooth to a plesiosaurian. However, the association between the tooth and the holotype specimen of *Janusaurus* is unclear. It may be a random association, but we feel this is unlikely given that this pattern has not previously been observed in any other specimens of ichthyosaurs or plesiosaurians in the Slottsmøya Member. The tooth could possibly be evidence of scavenging on the carcass by a plesiosaurian, however this also seems improbable given that the extremely gracile tooth morphology is suggestive of a soft-bodied invertebrate feeder [37]. Finally, there is a possibility that the tooth could represent gut contents of the ichthyosaur, although the diminutive tooth morphology of the ichthyosaur is seemingly inconsistent with this interpretation, because no other elements of a plesiosaurian were associated with the skeleton.

**Ontogeny**: The specimen is interpreted to be an adult, based on the smoothness of the humeral shaft, the concavity of the distal facets and the advanced ossification of the forefin elements [38], [39]. In addition, the cross-section shape of the dorsal ribs exhibit a distinct figure-eight shape with thick cortical bone, which Kear & Zammit [39] suggested as an ontogenetic adult trait. Interestingly, the humeri of PMO 222.654 possess flat proximal ends, which has been described as an indicator of immaturity [38]. However, this character may not be universally reliable given the specimen's size and other evidence of advanced ossification typical of other ophthalmosaurids [40], [41].

#### Dermatocranium

The skull was exposed near the surface, and much of the left side is missing or damaged. The skull is obliquely dorsolaterally compressed, and the chondrocranium is displaced and partially disarticulated. Most of the interpretations presented here are based on the better preserved right side of the skull, which remains largely articulated, although fragmented. However, in many cases bones of the skull can be confidently delimited on the basis of well-defined sutural margins. Cranial ratios classically employed in ichthyosaur taxonomy are not possible to present here because the rostrum and lower jaw are incomplete. A detailed photogrammetry video of the skull is available in Video S1.

The **premaxilla** is highly eroded, and its contact with the lacrimal is poorly preserved. However, the posterior-most portion of the subnarial process appears to contact and possibly overlap the lacrimal, ventral to the external naris. The posterior-most margin of the subnarial process closely approaches the jugal, but whether they contact is unclear (Figure 4). The premaxilla-nasal contact in the area anterior to the external naris is heavily eroded, and the suture is difficult to identify; as a result, the presence and/or nature of a supranarial process is uncertain. The right **maxilla** has considerable exposure along the lateral surface of the skull. Only the anterior-most tip of the maxilla is not preserved, but the element extends at least as far as the anterior end of the preserved rostrum. Its posterior end terminates just anterior of the midpoint of the ventral margin of the orbit. The maxilla is clearly discernable from the jugal along a clear sutural margin. However, the degree to which it extends dorsally, in the vicinity of the lacrimal and external naris, is unclear. So far as can be discerned, the maximum dorsoventral height of the maxilla lies in line with the posterior margin of the external naris. A dental groove in the premaxilla and maxilla holds small conical teeth.

Most of the right and posterior portion of left **nasal** is preserved. There is no sign of an internasal foramen, although this area is poorly preserved. The nasal forms the entire dorsal margin of the external naris and the prefrontal, excluding the latter from contact with the external naris. Dorsal to the external naris, the nasal projects laterally to form a prominent, though somewhat crushed, shelf. Posteriorly, the nasals fan out to overlap the postfrontal.

The **lacrimal** is robust, and its margins are easy to discern. The lacrimal forms the entire posterior and much of the ventral margin of the external naris. The jugal borders most of the ventral margin of the lacrimal save the anteroventral-most portion of the lacrimal. The lacrimal articulates with the prefrontal along a zone of well-defined interdigitating processes. As seen in lateral view, the posterior margin of the lacrimal is bent at an angle of approximately 140 degrees. Similar to the condition in other ophthalmosaurids, the lacrimal bears a prominent lateral ridge in the center of the element that extends from its posteroventral margin dorsally to near the border of the external naris [31]. The shape of the external naris can be somewhat discerned and is anteroposteriorly shorter than tall.

The **jugal** forms the entire ventral margin of the orbit. The anterior portion of the jugal markedly thins dorsoventrally, anterior to the orbit, and overlaps the ventral margin of the lacrimal. The anterior portion of the jugal is straight and is bordered ventrally by the maxilla. The right jugal is broken and distorted ventral to the mid-point of the orbit, but appears to gently curve dorsally along the posterior third of its length before contacting the postorbital (Figure 4).

The right **prefrontal** forms approximately one third of the dorsal margin of the orbit. The anterior edge of the prefrontal does not appear to contribute to the external naris. Together with the postfrontal, the prefrontal forms the supraorbital flange. The nasal and postfrontal overlap the prefrontal medially and posteriorly, respectively. The right **postfrontal** is a large and prominent element, whose size and extent are discerned on the basis of bone fiber orientation, which radiates from the center of the bone. It forms the posterior two-thirds of the dorsal border of the orbit. The nasals appear to overlap the anterior margin of the postfrontal. The postfrontal-postorbital contact is short externally, but the two elements share a long overlapping contact ventrally, on the medial side of the orbital rim (Figure 5). The postfrontal has a broad contact posteriorly and medially with the supratemporal, thereby excluding the postfrontal from participating in the lateral margin of the temporal fenestra. However, the postfrontal forms most, if not all, of the anterior border of the supratemporal fenestra. Medially the relationship of the postfrontal to the frontal is unclear. Anterior to the supratemporal fenestra, the postfrontal has a long, straight overlapping contact with the parietal that extends nearly to the midline of the skull.

The relationships of the **frontals** cannot be discerned due to poor preservation of the skull roof. However, part of the right frontal could be preserved at the dorsal-most area of the preserved portion of the skull, associated with a structure that could represent part of the pineal foramen. The right **parietal** is well preserved and forms the entire medial margin of the supratemporal fenestra. The parietal-supratemporal contact is hard to discern (Figure 5), although a supratemporal process is present. There is no indication of ornamentation or a sagittal crest along the dorsal surface of the skull. The supratemporal fenestra is anteroposteriorly longer than wide, although crushing makes it difficult to interpret its original shape.

The right **supratemporal** is exposed on both sides of the specimen. Its anterior and medial processes form all of the lateral and most of the posterior margins of the supratemporal fenestra, respectively. A **squamosal** was not identified in PMO 222.654, and is presumed to have been absent, as the region in which this element is usually present is well preserved in the specimen. The **postorbital bar** of *Janusaurus* is relatively narrow (Table 1) and has a postorbital bar ratio (maximum anteroposterior width versus anteroposterior length of the orbit) of 0.68. The **postorbital** has clear sutural relationships with the postfrontal dorsally, the supratemporal posterodorsally, the quadratojugal posteriorly, and the jugal ventrally. The **quadratojugal** contacts the postorbital along most of its posterior border and has a short underlapping contact with the supratemporal dorsally. As seen in lateral view, the ventral portion of the quadratojugal is anteroposteriorly broader than the dorsal half and thus projects caudally from the posterior margin of the postorbital bar.

**Table 1:** Selected cranial measurements of PMO 222.654 (in mm).

Preserved skull length (Postorbital to preserved anterior of premaxilla)	475
Anteroposterior length of orbit	220
Maximum dorsoventral hight of orbit	160
Anteroposterior length of postorbital bar	45
Preserved length of lower jaw (right)	520
Preserved jugal length	270
Preserved maxilla length	260
Lacrimal dorsal-ventral height	80

#### Braincase

PMO 222.654 preserves the basioccipital, basisphenoid and right stapes, but other elements are either not visible or not preserved. Selected measurements can be found in Table 2. The basioccipital of PMO 222.654 was disarticulated from the braincase but is largely intact, though fragmented. The element was not found in place and is poorly preserved. Our orientation is presented in Figure 6 A-D. The occipital condyle is intact, but the extracondylar area is eroded and part of it is missing. The occipital condyle is convex and is mediolaterally wider than tall. In dorsal and posterior view the condyle blocks the extracondylar area almost entirely from view, although an indication of the lateral facets for the ophisthotics are visible. It is only possible to describe the facet for the left ophisthotic because the right has been deformed. The length of this facet is approximately 3 cm. Most of the dorsal surface is damaged, but an indication of the left exoccipital facet is preserved. The anterior surface is fragmented, with little surface available for description. However, the preserved anterior surface is uneven and pitted and has a projection in the center of the anterior surface; this process extends ventrally and ends in an eroded “spine”. There is no indication of a ventral notch, but this area has been severely damaged, so this structure may have been present.

**Table 2:** Selected braincase measurements of PMO 222.654 (in mm).

Occipital condyle width/posterior width of basioccipital	9
Mediolateral width of basioccipital/	14
mediolateral height of basioccipital	
Maximum lateral length of basioccipital	60
Maximum lateral height of basioccipital	56
Length of preserved left ophistotic facet	30
Length of preserved left exoccipital facet	23

Only the right stapes was recovered from the dorsal part of the skull (Figure 6 E-H). The stapedial shaft is thin, rounded and gracile along the entire shaft, expanding slightly at the quadrate facet. The posterior side of the medial surface of the stapes is flat, and expands posteriorly from the medial head in dorsal view. The anterior margin is convex, especially at the medial stapedial head. A ridge on the articular surface of the medial stapedial head separates the surface for the ophisthotic from the ventrally-situated surface for the basioccipital and basisphenoid. This surface terminates in a ridge that could be homologous to the hyoid process described for *Ophthalmosaurus icenicus* and *Acamptonectes densus* [23], [31]. The entire medial stapedial head is rugose, and a tubercle on the ventral side is interpreted to be a surface for attachment of a hyoid ligament as in *O. icenicus* and some specimens of *Acamptonectes* [23]. The **basisphenoid** is lodged inside the orbit and was not possible to remove for description.

#### Palatal complex

The **palate** is poorly preserved, and most of the elements are lost. Parts of the posterior ramus of the right **pterygoid** are visible in medial view (Figure S1). The right pterygoid is displaced and damaged in several places. The quadrate ramus is drawn out into three processes, extending laterally, medially and dorsally. The ventral surface of the quadrate ramus is smooth, but with a more irregular posterior end. The pterygoid extends anteriorly up to the mid-point of the orbit, where it is eroded. The right pterygoid is visible but situated on top of the right quadrate, blocking it from medial view (Figure 7). The right **quadrate** is partially visible ventral to the postorbital and quadratojugal, and in posteromedial view beneath the right pterygoid (Figure S1). Unfortunately most of this element is not visible enough to describe in detail.

#### Mandible

PMO 222.654 only preserves an incomplete right lower jaw, visible in both lateral and medial views, but is missing its anterior end. Measurements of the individual elements can be found in Table 3. Only the posterior-most region of the mandible is well-preserved, particularly in medial view (Figure 7). The posterior margin of the **dentary** lies in line with the anterior third of the orbit. A small portion of the dental groove is visible in which diminutive conical teeth are held. The **surangular** extends at least as far anteriorly as the preserved portion of the jaw. In medial view, a small coronoid process is visible. Posterior to the coronoid process is a second and more prominent, dorsally-projecting process on the surangular, here termed the preglenoid process. This structure, whose entire medial surface is marked with ridges, has been interpreted to be the point of insertion for the M. adductor mandibulae externus group (MAME) in *Ophthalmosaurus* by Kirton [31] or the M. adductor mandibulae internus pseudotemporalis (MAMIP) by Kear [42].

**Table 3:** Selected mandibular measurments from PMO 222.654 (in mm).

Preserved angular length	485
Preserved suranglar length	453
Maximum posterior angular height	50
Maximum posterior surangular height	25
Approximate articular anteroposterior length	65
Height of preglenoid process	12
Height of coronoid process	2

In lateral view, the anterior end of the **angular** lies near the preserved portion of the skull. The angular contributes approximately two-thirds of the dorsoventral height at the posterior end of the mandible in lateral view. Although the posterodorsal portion of the ramus was lost during preparation, its size and shape can be determined by facets on the articular. In medial view, the angular extends further posteriorly than the prearticular and forms the ventral margin of the articular. The **prearticular** is dorsoventrally tall anteriorly as seen in medial view. The shape of the dorsal margin of the prearticular somewhat mirrors that of the surangular, in possessing a slight dorsal process before abruptly tapering in height posteriorly to a narrow process, and terminating along the anteromedial margin of the articular. Only a small portion of the **splenial** is preserved medially, but little can be said regarding its morphology.

The **articular** is articulated to the prearticular and angular, and enclosed behind the surangular in lateral view (Figure S2 L). It appears more anteroposteriorly elongated than round. The clear sutures for the prearticular on the medial side as well as for the surangular on the lateral side, suggest that the element is articulated. In medial view the articular is convex, bulging outwards posterodorsally, with a prominent ridge located along the posterior edge. The anterodorsal edge articulating with the quadrate is rounded and rugose, suggesting large amounts of connective tissue. The ventromedial side is smooth and is slightly concave, where it articulates with the prearticular and the angular. An articulation between the articular and the angular has not been previously identified in ophthalmosaurids, but occurs in some specimens of *Stenopterygius* [43]. Anterolaterally the articular is covered by the surangular and the angular. The medial surface of the articular is smooth in the center, becoming more irregular towards the articular surfaces.

#### Dentition

In medial view, disarticulated tooth fragments are visible. The total number of fragments preserved is uncertain, although they have remained in the jaw. The crown height based on partial fragments is very short with an estimated height less than 9 mm. Fine ridging is occurs on all sides of the teeth.

#### Scleral elements

A partially articulated but poorly preserved scleral ring is present in the right orbit. The minimum length of a single plate, as measured from the inner to outer margin of the ring, is approximately 6 cm.

#### Hyoid apparatus

A pair of **hyoid** rods was found on the ventromedial side of the angular, but only the right hyoid was in sufficient condition to be prepared (Figure 8). The rod is completely three dimensional and gently curved in shape. The anterior end is convex and club-like. The shaft of the hyoid rod is rounded in cross section and its posterior end is semi-spatulate.

### The appendicular skeleton

#### The pectoral girdle

The pectoral girdle of PMO 222.654 was articulated, although slightly displaced, and lacks only the left scapula (Figure 9). The clavicles are well-preserved. The right clavicle is 1.5 times as long as the anteroposterior length of the right coracoid (Table 4; Figure 9 A-D). The medial ends of both clavicles end in finger-like projections that neatly interdigitate with their opposites [44]. The visceral surfaces of the medial portions of the clavicles are dished, so as to envelope the lateral rami of the interclavicle. The lateral ends of the clavicles curve dorsally and posteriorly and bear facets for the scapula. The interclavicle is complete and well preserved (Figure 9 E-F). The lateral rami are dorsoventrally flattened and ventrally convex for reception with the clavicles. The combined mediolateral width of both lateral rami is greater than the anteroposterior length of the interclavicle. Located on the anterior half of the dorsal surface of the posterior ramus are two dorsally-projecting processes that form the lateral margins of a narrow, anteroposteriorly-oriented excavation, which is here termed the interclavicluar trough. Not all of the shale imbedded in this posterior-most section of this trough was removed during preparation. Thus, the true posterior extent of the interclavicular trough is longer than shown on Figure 9 E-F. The posterior end of the element is dorsoventrally flattened and is somewhat expanded mediolaterally. A foramen is located on the ventral surface of the interclavicle, where the lateral and posterior rami meet.

**Table 4:** Selected pectoral girdle measurements of PMO 222.654 (in mm).

Coracoids		
*Coracoid Left*		
	Maximum mediolateral width	201
	Maximum anteroposterior length	206
	Length of intercoracoid suture	142
*Coracoid Right*		
	Maximum mediolateral width	213
	Maximum anteroposterior length	219
	Length of intercoracoid suture	156
	Length of scapular facet	45
	Length of glenoid facet	49

Scapula (Right)		
	Maximum proximodistal length	173

Clavicles		
*Clavicle left*		
	Preserved maximum length	306
	Maximum width	75
*Clavicle right*		
	Maximum length	344
	length of scapula facet	45
	Maximum width	76
Interclavicle		
	Preserved anteroposterior length	160
	Maximum length of lateral bar	219

The coracoids (Figure 9 H) are slightly longer anteroposteriorly than wide mediolaterally. The medial symphysis forms the anterior two-thirds of the coracoid; in medial view the outline of the symphysis is lenticular and markedly short dorsoventrally. In dorsal view, the anterior margin of the coracoid forms a prominent anteromedial process, which marks the lateral margin of a well formed anterior notch. The scapular and humeral facets are approximately equal in size and are offset at an angle of 150 degrees from the sagittal plane. Only a poorly-preserved and incomplete right scapula was recovered (Figure 9 G). The suprascapular border is intact, although rather short compared to the size of the rest of the pectoral girdle. The anterior portion of the scapular blade is distorted and folded, which precludes further description.

#### Forefin

Two partial forefins are preserved in PMO 222.654. The left forefin includes a complete humerus disarticulated from the pectoral girdle with an articulated zygopodium and a few associated autopodial elements. The right humerus was entirely disarticulated and was found in the vicinity of an isolated right radius. The orientation and identity of the humeri was determined by comparisons with articulated limbs of other ophthalmosaurid remains from the Slottsmøya Member and from published descriptions [15], [31], [45]. The anteroposterior axis was determined by the location of the preaxial accessory element and the dorsoventral orientation via the shape and position of the dorsal process and deltopectoral crest (Figure S2 A-D).

The **humerus** is proximodistally longer than anteroposteriorly broad at its distal end. The distal end is anteroposteriorly broader than the proximal end (Figure 10 A-B). The minimum anterior-posterior width at midshaft is 16% smaller than the maximum anteroposterior width of the proximal end, giving a length-to-width ratio of 1.8. The proximal articular surface of the humerus is relatively flat, with a rugose surface texture. An elevated rim circles the periphery of the dorsal, anterior and ventral portions of the articular facet. In dorsal view, the dorsal process originates near the anteroposterior midpoint of the articular facet and extends to midshaft. It is slightly angled towards the anterior margin. The dorsal process is relatively tall and narrow compared to the deltopectoral crest, which is more broadly rounded (Figure 10 E). The deltopectoral crest begins near the anterior margin of the humerus, extends to near the midpoint of the shaft, and slants slightly posteriorly. In anterior view, the preaxial margin of the humeral shaft is dorsoventrally shorter than tall and has a broadly rounded postaxial margin.

Distally, the humerus bears three concave distal facets. The anterior-most, for the preaxial accessory element, is approximately half the anteroposterior length and dorsoventral height of the radial facet (Table 5). The ulnar facet is dorsoventrally taller than the radial facet, but slightly shorter anteroposteriorly (Table 5). Relative to the long axis of the humerus, the facets for the preaxial accessory element and the ulna are angled at approximately 17 degrees anteriorly and 30 degrees posteriorly, respectively.

**Table 5:** Selected measurements from the left humerus of PMO 222.654 (in mm).

Maximum proximodistal length	152
Maximum anteroposterior width, proximal end	104
Maximum dorsoventral height, proximal end	86
Maximum anteroposterior width, distal end	136
Maximum dorsoventral height, proximal end	69
Mimimun anteroposterior width, midshaft	85
Length of radial facet	49
Length of ulnar facet	61
Length og preax. element facet	25

The zygopodial row includes the radius, ulna and a preaxial accessory element, where the radius and ulna are recognized as the two largest elements [31], [45]. The preaxial accessory element is oval-shaped in dorsal view, but is dorsoventrally narrow anteriorly. The proximal margin is three times the dorsoventral thickness of the distal end. The preaxial accessory element bears two distinct articular surfaces, one proximally for the humerus and the other posteriorly for the radius. The ulna and radius are similar in dorsoventral height, but the ulna is anteroposteriorly longer. In dorsal view, the ulna is triangular in outline, whereas the radius is more oval. Both elements are convex on their articular facets for reception by the humerus (Figure 10 A). The surfaces in contact with the humerus and other elements are pitted and rugose.

Very little of the autopodium of PMO 222.654 has been preserved (Figure 10 A). The left intermedium and possibly the left radiale are semi-articulated with the left zygopodium. The intermedium articulates snugly between the radius and ulna and is twice as thick dorsoventrally at its proximal end than at its distal end. Several other small elements were found in the vicinity of the left humerus but their identity is equivocal. It is not possible to determine the number of digits in PMO 222.654.

#### The pelvic girdle

PMO 222.654 includes two disarticulated ilia and an ischiopubis found in the vicinity of a series of articulated caudal vertebrae, along with both disarticulated femora and several limb elements (Figure 11).

One ilium is complete, whereas the other is missing its distal portion (Figure 11 A-E). Our preferred orientation for the ilia follows the description for *Ophthalmosaurus icenicus* provided by Kirton [31], comparisons with a complete and articulated pelvic girdle of *Cryopterygius kristiansenae* [15] and descriptions from other articulated specimens [46]. Determination of the mediolateral axis is based on curvature of the element along its proximodistal axis, with the concave surface being medial. We interpret the markedly concave margin of the ilial shaft to be facing posteriorly. The proximal end is mediolaterally flattened, and the proximal end twists medially, closer to the vertebral column. The distal end is slightly larger than the proximal end and has two facets for the ischiopubis and femur. Using these criteria we interpret the one complete ilium to be the right. Using this orientation, the prominent process visible on each ilium is located in the proximal half of the element and projects anterodorsally (Figure 11 A-D). This ilial process occurs on both elements and is not a taphonomic artifact. The ilial process ends in a blunt tip and curves slightly medially. The lateral surface of the process bears small ridges and the medial surface is rugose. Both the proximal and distal ends of the ilia are pitted.

The preserved **ischiopubis** is interpreted to be the right, based solely on its association with the right ilium. It is nearly complete but is slightly eroded at its distal end. The element is mediolaterally thickened proximally, but is otherwise very flat and anteroposteriorly broader distally (Figure 11 F-G). Both the ischium and pubis are fused along their entire length, and there is no indication of an obturator foramen. The maximum length is 3.3 times longer than the maximum width (Table 6). The proximodorsal edge bears a ridge that flattens towards the distal end [31].

**Table 6.** Selected pelvic girdle measurements of PMO 222.543 (in mm).

#### Hindfin

The hindfins are completely disarticulated and include two femora and several partial zygopodial and/or autopodial elements. These elements are too damaged to identify and describe. Measurements of the individual femora can be found in Table 7.

**Table 6:** Selected pelvic girdle measurements of PMO 222.543 (in mm).

Ilium		
	Maximum anteroposterior length	97
	Acetabulum process length	9
	Posterior height	22
	Length of anterodorsal process	24

Ischiopubis		
	Maximum proximodistal length	181
	Anteroposterior length, proximal end	35
	Anteroposterior length, distal end	55
	Dorsovental thickness proximal end	11

**Table 7:** Selected femora measurements of PMO 222.654 (in mm).

Left Femur		
	Maximum proximodistal length	103
	Maximum anteroposterior width, proximal end	57
	Maximum height, proximal end	49
	Maximum anteroposterior width, distal end	58

Right Femur		
	Length of tibial facet	17
	Length of fibular facet	21

The identity of the femora was determined using the articulated pelvic girdle and hind fin of *Cryopterygius* [15] and Maxwell et al. [47]. The left **femur** (Figure 12) is better preserved than the right (Figure S2 E-J), but the distal ends of both femora are compressed, so the facets for tibia and fibula are unclear. The proximal and distal ends are nearly identical in anteroposterior width. The proximal articular surface is well preserved in the left femur. This surface is convex dorsally and concave ventrally at the location of the ventral process. In proximal view, the femur is approximately as dorsoventrally tall as anteroposteriorly wide. In ventral view, an anteroposteriorly broad ventral process extends to near the midpoint of the femur. The dorsal process originates near the anterior margin and terminates midway along the shaft. There appear to be only two facets located on the distal end, although this is equivocal. On the right femur a slight ridge seems to separate the two facets (Figure S2 H). The fibular facet is approximately 20 percent longer than the tibia facet. The anteroproximal side appears to have several foramina.

#### The axial skeleton

A total of 21 complete to partially complete vertebrae were found, including the atlas-axis, four articulated cervicals and several dorsal vertebrae, which were associated with the skull. Seven articulated caudal vertebrae were found near the pelvic girdle, along with 10 other disarticulated sacral/caudal vertebrae. The **atlas-axis** complex is incomplete and is laterally compressed, with only the right lateral side (stratigraphically up) preserved. The complex is completely fused and lacks any sign of a suture. The anterior face of the atlas (Figure 13 A-B) is broad compared to the axis, and more deeply cupped to articulate with the occipital condyle. Most of the dorsal and cervical vertebrae were left in articulation with the dorsal ribs, but a single **cervical vertebra** was removed, directly posterior to the atlas-axis (Figure 13 C-F). It is laterally compressed and deformed, but facets for the right rib and right side of the neural arch are visible. The anterior surface of the vertebrae is very concave and slightly irregular, suggesting connective tissue investment. Two partial and disarticulated **neural arches** from the sacral- and trunk region, were preserved but cannot be measured or described further due to poor preservation (Figure 13 G-H).

Most of the preserved **dorsal** **ribs** are associated with the dorsal vertebrae. The trunk ribs are estimated to be on average 90 cm in length. The most complete of these measures 84 cm in length, although approximately 10 cm of the distal end was missing (Figure 13 I). The ribs are figure-eight shaped in cross section along the entire length of the shaft, apart from the circular cross section at its ventral termination [46]. PMO 222.654 preserves a large number of **gastralia**, which are seldom described in ophthalmosaurids. The gastralia have a circular cross section. Their medial ends are softly rounded and the lateral ends terminate in a thin point (Figure 13 J).

## Discussion

*Janusaurus lundi* can be confidently placed in Ophthalmosauridae on the basis of having a reduced extracondylar area of the basioccipital, extensive lateral exposure of the angular, and a preaxial accessory element in the forelimb [46]. The specimen has an anterior twisting dorsal process on the humerus, which has also been proposed as a synapomorphy of the clade [48]. A broad contact between the premaxilla and lacrimal, although not present in *Platypterygius australis* [42], has been identified as a general synapomorphy, which the specimen could possibly share [40]. The following discussion compares *Janusaurus lundi* to all described Middle Jurassic to Early Cretaceous ophthalmosaurids as well as an undescribed ophthalmosaurid, PMO 222.667, from the Agardhfjellet Formation and *Malawania anachronus* [28]. A skull reconstruction can be found in Figure 14.

### Dermatocranium

The subnarial process of the premaxilla appears to have contacted and possibly even broadly overlapped the anterior process of the lacrimal, similar to that of *Caypullisaurus bonapartei* and *Aegirosaurus leptospondylus* [49]–[51], but unlike *Platypterygius australis* which lacks this contact [42]. Although a premaxilla-jugal contact is unclear, this area lacks any evidence for the broadly interdigitating contact seen in *Brachypterygius extremus* [52]. The subnarial process of the premaxilla of *Janusaurus lundi* participates in the anterior and anteroventral boundary of the external naris similar to the condition in *C. bonapartei* and *A. leptospondylus* [49]–[51], but unlike the morphology in *P. australis* and *Athabascasaurus bitumineus* which lack any contact [42], [53]. This also differs from the condition in *Ophthalmosaurus icenicus*, where the premaxilla only borders the anteroventral margin of the external naris [31].

The maxilla of *Janusaurus lundi* has considerable lateral exposure, particularly posteriorly, similar to the condition in *Caypullisaurus bonapartei*, *Palvennia hoybergeti* and *Cryopterygius kristiansenae* [15], [49], but not to the degree of *Leninia stellans*, where the maxilla extends as far as the mid-point of the orbit [29]. This trait is absent in *Ophthalmosaurus icenicus* and *Athabascasaurus*, where the maxilla has almost no posterior exposure [31], [53]. The maxilla of *J. lundi* fails to contact the lacrimal; this differs from *O. icenicus, Maiaspondylus lindoi* and *P. hoybergeti*, where the ventral border of the lacrimal clearly contacts the maxilla in lateral view [15], [31], [54]. The maxilla does not appear to clearly contact the external naris as in *Platypterygius australis* and *M. lindoi* [42], [54]. In the description for *M. lindoi*, Maxwell and Caldwell [54] described the extensive overlap between jugal and maxilla as an autapomorphy for the species, but this feature is now also described in *J. lundi* and *C. kristiansenae* [15]. Despite lacking its anterior-most margin, the maxilla shows extensive anterior and lateral exposure compared to most ophthalmosaurids [15], [42], [53].

The nasal of *Janusaurus lundi* participates in the external naris and forms the dorsal border, possibly as an arched overhang similar to the morphology in *Platypterygius australis*, and *Ophthalmosaurus icenicus* [31], [42]. The nasal forms the entire dorsal boundary of the prefrontal similar to the morphology in *O. icenicus, Sveltonectes insolitus* and *Palvennia hoybergeti*, but unlike the condition in *Athabascasaurus bitumineus* where the posterodorsal portion of the prefrontal is covered by the postfrontal [15], [31], [41], [53].

A prominent anterior process of the lacrimal is present and more pronounced than in Cryopterygius kristiansenae [15], but less pronounced than in Aegirosaurus leptospondylus, Ophthalmosaurus icenicus, Platypterygius americanus and Platypterygius bannovkensis [31], [51], [55], [56]. The lacrimal of Janusaurus lundi clearly forms the entire posterior border of the external naris, unlike the anatomy in C. kristiansenae, Platypterygius australis and Athabascasaurus bitumineus, where the lacrimal is excluded from the external naris by an ascending process from the maxilla [15], [42], [53]. The gently concave orbital margin of the lacrimal in J. lundi contrasts with the 90 degree bend seen in C. kristiansenae [15]. The external naris is dorsoventrally taller than anteroposteriorly long, similar to that in C. kristiansenae, but unlike the condition in other ophthalmosaurids such as Palvennia hoybergeti, O. icenicus and A. leptospondylus, where the naris is longer than tall [15], [31], [51]. The prefrontal does not participate in the border of the external naris, as in O. icenicus and Sveltonectes insolitus [31], [41].

There appears to be significant variation of the morphology and relationships of the anterior process of the jugal in ophthalmosaurids. Thus, we have separated the variation into two different states and incorporated them into the phylogenetic analysis (Character 11): 1) the anterior process of the jugal terminates posterior to the anteroventral margin of the lacrimal; 2) the anterior process of the jugal reaches or extends anterior to the anteroventral margin of the lacrimal. Most ophthalmosaurids possess the first state (0), including *Leninia stellans, Maiaspondylus lindoi*, *Cryopterygius kristiansenae*, *Caypullisaurus bonapartei*, *Aegirosaurus leptospondylus*, *Sveltonectes insolitus*, *Ophthalmosaurus icenicus* and *Athabascasaurus bitumineus* [15], [29], [31], [41], [51], [53], [54], while *Platypterygius australis, Caypullisaurus bonapartei and Brachypterygius extremus* have the second state (1) [31], [42], [50]. *Janusaurus lundi* is tentatively referred to the first state, as the jugal terminates ventral to the lacrimal but does not reach the anteroventral end. The shape of the jugal at the ventral border of the orbit also shows great variation throughout Ophthalmosauridae; the degree of “bowing” varies from being entirely straight, to being gently bowed. The jugal of *J. lundi* has a slightly bowed anterior process of the jugal which is neither as straight as the jugal of *B. extremus* and *C. kristiansenae*, or as gently bowed as the jugal observed in *A. leptospondylus, O. icenicus*, *Palvennia hoybergeti* and *P. australis* [15], [31], [42], [51], [52]. The variation in the degree of bowing needs to be further investigated and constrained considerably before being included in a phylogenetic analysis. The presence of a posterodorsal process of the jugal articulating with the postorbital is also subject to variation across the majority of described species in which this region is well-preserved, including *O. icenicus*, *A. leptospondylus*, *P. australis*, *S. insolitus* and *A. bitumineus* [31], [41], [42], [51], [53]. In *C. kristiansenae* the process is absent entirely, whereas in *J. lundi* the process is significantly prominent, comprising nearly half of the posterior margin of the orbit [15]. There is also great variation in the elements contacting the posterodorsal process of the jugal. We have separated this variation into two states, which are included into the phylogenetic analysis (Character 12): 1) the posterior margin of the jugal articulates with the quadratojugal and the postorbital; 2) the quadratojugal is excluded from the posterior margin of the jugal by the postorbital. In *J. lundi*, *S. insolitus* and *A. bitumineus* the quadratojugal is excluded from the jugal by the postorbital [41], [53]. Alternatively, in *O. icenicus* and *Leninia stellans* the jugal is overlapped by the postorbital, but a posterior flange overlaps the quadratojugal [29], [31], and in *C. kristiansenae*, *P. australis* and *A. leptospondylus*, the jugal borders the postorbital and quadratojugal ventrally [15], [42], [51].

The anteromedial edge of the postfrontal overlaps the parietal broadly, possibly similar to the state in *Athabascasaurus bitumineus* [53] but unlike the postfrontal in *Ophthalmosaurus icenicus*, where this is limited to a small area at the anterior of the supratemporal fenestrae [31]. The posterior process of the postfrontal expands mediolaterally to overlap the supratemporal, unlike the condition in *Aegirosaurus leptospondylus*, *Platypterygius australis* and *Leninia stellans*, where the postfrontal is constrained and terminates narrowly posteriorly [29], [42], [51]. There is no obvious parietal crest or ridge, unlike *P. australis*, where a parietal crest is present [42].

The extent of the lateral margin of the supratemporal of *Janusaurus lundi* resembles that in *Athabascasaurus bitumineus*, forming the entire lateral margin of the supratemporal fenestra. This condition differs from that in *Aegirosaurus leptospondylus*, *Ophthalmosaurus icenicus* and *Platypterygius australis*, where the supratemporal forms only half of the border [31], [42], [51]. The supratemporal forms most of the posterior border of the supratemporal as in most ophthalmosaurids, which differs from the reduced condition in *Leninia stellans* [29].

The postorbital bar is relatively narrow anteroposteriorly, similar to that of *Palvennia hoybergeti*, *Aegirosaurus leptospondylus*, *Nannopterygius enthekiodon* and *Ophthalmosaurus icenicus* [15], [31], [51], but markedly different from the broad configuration observed in *Cryopterygius kristiansenae, Caypullisaurus bonapartei* and *Platypterygius americanus* [15], [49], [55]. The postorbital of *Janusaurus lundi* has reduced lateral exposure compared to that in *Platypterygius australis*, *A. leptospondylus*, *Sveltonectes insolitus* and *Athabascasaurus bitumineus* [41], [42], [51], [53]. The quadratojugal has a large lateral exposure, more than in *O. icenicus* but not to the degree of *A. bitumineus* [31], [53]. Similar to the anatomy in *O. icenicus* and *Platypterygius australis*, the quadratojugal of *J. lundi* bears a short posterior process [31], [42], which is absent in *C. kristiansenae* and *A. bitumineus* [15], [53]. The absence of a squamosal in *J. lundi* is similar to the condition in *P. hoybergeti* and *C. kristiansenae* [15], but unlike the condition in *O. icenicus, Brachypterygius extremus* and *A. bitumineus* [31], [52], [53].

The extracondylar area of the basioccipital in *Janusaurus lundi* is reduced as in *Palvennia hoybergeti, Sveltonectes insolitus, Aegirosaurus leptospondylus, Arthropterygius chrisorum* and PMO 222.667 [15], [33], [40], [41], [51]. This contrasts with the condition seen in *Ophthalmosaurus icenicus*, *Mollesaurus periallus* and *Caypullisaurus bonapartei*, where the extracondylar area is more prominent [31], [49], [50], [57]. In posterior view, the outline of the basioccipital differs markedly from the more pentagonal shape seen in *O. icenicus*, *M. periallus* and the undescribed PMO 222.667 [31], [57], [58], by being mediolaterally wider than dorsoventrally tall, giving it an oval-like shape similar to that seen in *Athabascasaurus bitumineus, S. insolitus, Palvennia hoybergeti* and *Platypterygius* spp.[15], [41], [42], [53]. In lateral view, the profile of the basioccipital is similar to *P. hoybergeti* in being anteroposteriorly longer than tall. The posterior face appears to lack a notochordal pit present in *A. chrisorum* and *P. hoybergeti* [15], [40]. *J. lundi* lacks a ventral notch, which is present in *O. icenicus* [31].

The gracile stapes of *Janusaurus lundi* differs from the stouter, more massive stapes of *Ophthalmosaurus icenicus, Mollesaurus periallus, Athabascasaurus bitumineus* and *Platypterygius australis* [31], [42], [53], [57], and its stapedial shaft is even thinner than that of *Palvennia hoybergeti* and *Acamptonectes densus* [15], [23]. The quadrate process is reduced, similar to *A. densus* [23]. The horizontally-oriented ridge on the medial surface separates the facet for the ophisthotic and that for the basioccipital and basisphenoid, unlike the groove described in *P. hoybergeti* and *O. icenicus* [15], [31]. A depression is also described in *A. densus*, but is situated anteroventrally [23]. The articular face for the ophisthotic is relatively smaller in *J. lundi* than that observed in *P. hoybergeti* [15].

The angular comprises two-thirds of the dorsoventral height of the posterior ramus of the mandible of *Janusaurus lundi*, similar to the angular in *Athabascasaurus bitumineus* [53]. This differs from the condition in other ophthalmosaurids, such as *Ophthalmosaurus icenicus* and *Palvennia hoybergeti*, where the posterior ends of the angular and surangular contribute equally to the dorsoventral height of the ramus in lateral view [15], [31]. The apparent contact between the angular and the articular, which is not observed in other ophthalmosaurids, has been described in some species of *Stenopterygius* [43].

*Janusaurus lundi* has a prominent dorsomedial preglenoid process on the surangular, which has been described in some specimens of *Ophthalmosaurus icenicus*, *Platypterygius australis* and *Acamptonectes densus* (GLAHM 132588) [23], [31], [42]. A low coronoid process occurs just anterior to the preglenoid process of the surangular, which is also present in *O. icenicus*. A similar preglenoid process and a coronoid process can be observed on a specimen of *O. icenicus* (OUM J.10574/12) and similar, although less distinct processes are seen on specimen PMO 222.654. No such processes occur in *Palvennia hoybergeti* [15]. A slight dorsal process is present also on the prearticular, which is a putative autapomorphy of this species, as this element is usually covered by the splenial in other species.

Recent work has demonstrated that the dentition of ophthalmosaurids can vary considerably in size and in the number of teeth [15], [20], [41], [53], [59]. The fragmentary teeth found in the holotype specimen of *Janusaurus lundi*, appear smaller and more gracile compared to those in other ophthalmosaurids, such as *Cryopterygius kristiansenae*, *Palvennia hoybergeti*, *Brachypterygius extremus* and *Platypterygius australis* [15], [31], [42]. Poor preservation precludes further comparisons.

Hyoid elements are infrequently reported in ophthalmosaurids, in part because they are usually disarticulated or obscured from view [46]. Hyoids have been described in specimens of *Sveltonectes insolitus*, *Platypterygius* spp., *Acamptonectes densus*, and *Cryopterygius kristiansenae* (*pers. obs.* AJR, PMO 214.578) [15], [23], [41], [42]. In all specimens, the hyoids are rod-like and slightly curved. The anterior end is convex, club-like, smooth and laterally compressed in in PMO 222.654, as in *S. insolitus*, whereas the posterior end is convex and club-like in *Platypterygius australis* and *Platypterygius hercynicus* ([41], [42], [60].

### Appendicular and axial skeleton

The taxonomic utility of the pectoral girdle in many ophthalmosaurids is questionable, due to the large amounts of intraspecific variation [61]. However, the pectoral girdle of *Janusaurus lundi* is well preserved and provides new data on morphological variation in this region. The clavicles of *J. lundi* are more robust and thickened in comparison to those in *Cryopterygius kristiansenae* and PMO 222.667 [15], and more closely resemble those of *Ophthalmosaurus icenicus* and *Aegirosaurus leptospondylus* [31], [51].

The scapula of *Janusaurus lundi* is notably shorter proximodistally than the same element in most ophthalmosaurids, when compared against the anteroposterior length of the coracoid, a feature that does not seem attributable to taphonomic distortion. The interclavicles of *Ophthalmosaurus icenicus* (*pers. obs*. AJR: NHMUK R.2180, R.2853, R.4753 CAMSM; J.68689eb, OUM; J.48012) and PMO 222.667 are conspicuously thickened along the anterior margin of the lateral ramii; this thickening is absent in *J. lundi*. Most notably the presence of an interclavicular trough is unknown in any other ophthalmosaurid, and this appears to be an autapomorphy of *J. lundi*. Likewise, a ventral foramen is absent in *O. icenicus*, *Aegirosaurus leptospondylus* and *Cryopterygius kristiansenae* [15], [31], [51].

The coracoids are similar in shape to that of *Acamptonectes densus, Ophthalmosaurus icenicus* (*pers. obs*. AJR CAMSM J29808, OUM J.48009), *Cryopterygius kristiansenae* and *Aegirosaurus leptospondylus*, and are most similar to that of *Arthropterygius chrisorum* and *Undorosaurus gorodischensis* in general morphology [15], [23], [30], [40], [44], [51]. They differ from the strongly dorsoventrally thickened coracoids in *Platypterygius australis* and *Sveltonectes insolitus* [41], [62]. The intercoracoid symphysis extends along two-thirds of the medial length in *Janusaurus lundi*, in contrast to *C. kristiansenae*, where the symphysis forms only half of the medial length [15]. The coracoid symphysis of *J. lundi* is dorsoventrally thinner than in *S. insolitus*, *O. icenicus* and *P. australis* and is of similar height to that of *A. chrisorum* [31], [40], [41], [62]. The anterior notch in *J. lundi* is rounded and shallow, in contrast to *S. insolitus*, where it is deep and narrow, and in *C. kristiansenae* and *A. densus* where it is deep and wide [15], [23], [41]. The scapular facet is approximately half the size of the glenoid facet as in *A. chrisorum* and *C. kristiansenae* [15], [40], in contrast to the somewhat smaller facet in *U. gorodischensis* [30].

In overall morphology the humerus of *Janusaurus lundi* is most similar to that in *Arthropterygius chrisorum*, *Undorosaurus gorodischensis* and *Maiaspondylus lindoi* [30], [40], [54], in that there is little constriction of the midshaft and similar anteroposterior lengths on the proximal and distal ends. This differs from the humerus of *Cryopterygius kristiansenae*, *Sveltonectes insolitus* and *Platypterygius australis*, where the proximal end is enlarged or of the same length as the distal end [15], [41], [62]. Similar to the humerus in the holotype specimen of *A. chrisorum* and to some extent that in *S. insolitus* [40], [41], the proximal surface of the humerus in *J. lundi* is nearly flat, even though these specimens are interpreted to be adults. The elevated rim found on the proximal articular surface of *J. lundi* and PMO 222.667 is similar to the condition described for *U. gorodischensis* and *A. chrisorum*. However, pending first-hand examination of the specimens, it is unclear if these structures are truly homologous [30], [40]. The short and narrow dorsal process of *J. lundi* differs from the tall dorsal process found in *S. insolitus*, *Acamptonectes densus* and many species of *Platypterygius* [23], [41], [60], [62]. Unlike the humerus of *Ophthalmosaurus icenicus* [31], [46], the deltopectoral crest of *J. lundi* is more prominent in proximal and anterior views, although it is still relatively shorter than the dorsal process.

There are three large and distinct distal facets on the humerus in *Janusaurus lundi*, while only two distal facets are found in *Nannopterygius enthekiodon*, *Platypterygius americanus* and *Sveltonectes insolitus* [31], [41], [63], and a diminutive third facet is sometimes found in *Cryopterygius kristiansenae* and *Undorosaurus gorodischensis* [15], [30]. In some taxa which possess three facets, such as *Brachypterygius extremus*, *Aegirosaurus leptospondylus* and *Maiaspondylus lindoi* [51], [52], [54], the anterior-most is interpreted as the radius, not the preaxial element, as found in *J. lundi*. The facet for the radius is dorsoventrally wider, where the facet for the ulna is anteroposteriorly longer, unlike *Arthropterygius chrisorum* and *Ophthalmosaurus icenicus*, where the radius facet is the larger [31], [40]. However, this trait seems to vary individually, particularly in *Acamptonectes densus* [23].

The elements of the zeugopodium of the forefin in *Janusaurus lundi* vary in dorsal outline; the radius is oval, the ulna slightly triangular and the preaxial element is circular. This differs from the elements in *Sveltonectes insolitus*, *Cryopterygius kristiansenae*, *Aegirosaurus leptospondylus* and *Maiaspondylus lindoi*, where the radius and ulna are pentagonal in form [15], [41], [51], [54]. The preaxial accessory is three times thicker proximally than distally as in *Arthropterygius chrisorum* and *Ophthalmosaurus icenicus* [31], [40]. The radius is slightly convex on the proximal surface, similar to the radius in *O. icenicus* [31]. The ulna is the largest element in PMO 222.654, as in specimens of *O. icenicus* and *S. insolitus* [31], [41]. The pointed process observed on the proximal edges of the ulna and radius in *A. chrisorum* are not present on the described specimen [40].

The pelvic girdle of *Janusaurus lundi* is distinctive compared to that in all other ophthalmosaurids, in that the ilium bears a prominent, anterodorsally directed ilial process. A pelvic element figured in the description of *Caypullisaurus bonapartei*, is tentatively interpreted as a pubis [49], [50]. Given the unusual ilial morphology seen in *J. lundi*, there is the possibility that this element may in fact be an ilium. The described specimen PMO 222.654 illustrates the possible morphological variation of this element, so the pelvic element of *C. bonapartei* should be reexamined to confirm this.

The ischiopubis of *Janusaurus lundi* lacks an obturator foramen, similar to the condition in *Aegirosaurus leptospondylus*, *Athabascasaurus bitumineus*, *Platypterygius australis* and *Sveltonectes insolitus* [41], [51], [53], [64]. Many other ophthalmosaurids possess an obturator foramen, such as *Ophthalmosaurus icenicus* [31], whereas *Cryopterygius kristiansenae* and *Undorosaurus gorodischensis* have an incompletely fused ischium and pubis at their distal ends [15], [30]. In *J. lundi*, the distal end of the ischiopubis is 1.6 times wider than the proximal end, whereas it is over twice as wide in *A. leptospondylus* [51].

The femur of *Janusaurus lundi* measures two-thirds of the proximodistal length of the humerus, as in *Arthropterygius chrisorum* and *Ophthalmosaurus icenicus* [31], [40], but the femur is only half the size of the humerus in *Caypullisaurus bonapartei*, *Platypterygius hercynicus* and *Aegirosaurus leptospondylus* [49]-[51], [60].The proximal and distal ends have the same maximum anteroposterior width, unlike the femur in *Cryopterygius kristiansenae* and *Undorosaurus gorodischensis* where the distal end is more expanded [15], [30]. The proximal end of the femur in *Platypterygius australis* differs from the condition in *J. lundi*, in that the dorsal and ventral processes are both prominent, giving it a completely different shape [64]. The dorsal process is small and rounded in profile and is located anteriorly as in *O. icenicus*, *A. chrisorum* and *C. kristiansenae* [15], [31], [40]. In contrast, the dorsal process in *Sveltonectes insolitus* and *P. australis* is much narrower and is located closer to the proximal-distal midline of the femur in *P. australis* [41], [64]. *J. lundi* has a prominent ventral process on the femur, similar to *A. chrisorum* and *C. kristiansenae* [15], [40]. The ventral process of *J. lundi* terminates just beyond the mid-point of the femur, as in *S. insolitus* and *Platypterygius americanus* [41], [55], but unlike *O. icenicus*, *C. kristiansenae* and *A. chrisorum* where it terminates at or proximal to the mid-point [15], [31], [40]. As in most ophthalmosaurids, the distal end of the femur in *J. lundi* bears two facets for the tibia and the fibula, in contrast to *Platypterygius spp*., which have three [63].

### Phylogeny

**Figure 15:**
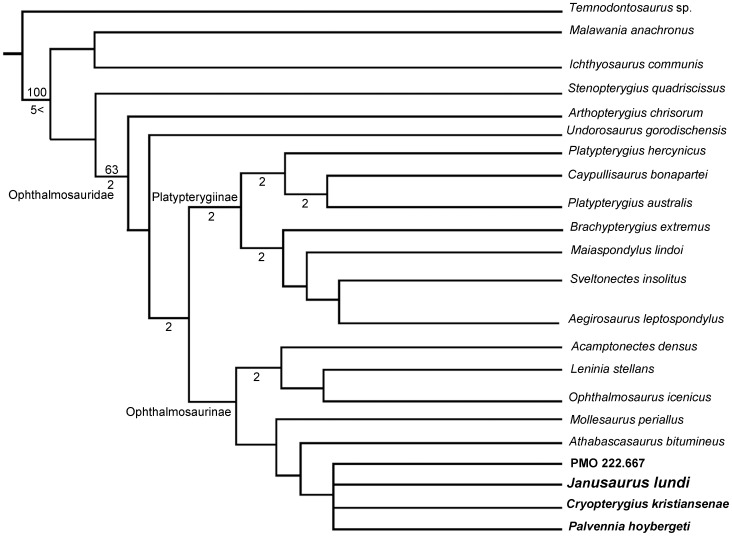
Strict consensus tree of the family Ophthalmosauridae. The tree is formed of three MPTs with 128 steps, showing relationships among Ophthalmosauridae and the outgroups. Boot strap value marked with number for values >50%, Bremer Support values shown for 2 and above.

Our analysis resulted in 3 equally most parsimonious trees, with a length of 128 steps; the strict consensus tree is presented in Figure 15. Congruent with Fischer et al. [23], *Arthropterygius* is recovered as the basal member of Ophthalmosauridae, along with *Undorosaurus*, which is included here for the first time in a phylogenetic analysis. Our analysis also recovers two major ophthalmosaurid clades, that are broadly congruent with Ophthalmosaurinae and Platypterygiinae of Fischer et al [23]. Within Platyperygiinae, the tree topology is largely similar to that recovered by Fischer et al. [23], with the major exception being the exclusion of Athabascasaurus, which is here nested in Ophthalmosaurinae. In our analysis, Ophthalmosaurinae is greatly expanded from that recovered by Fischer et al. [23] in terms of taxonomic composition. Within Ophthalmosaurinae, there are two subclades; 1) *Acamptonectes densus* (*Leninia stellans*+*Ophthalmosaurus icenicus*), and 2) *Mollesaurus periallus* (*Athabascasaurus bitumineus* (SML taxa)). However, it is noted that bootstrap percentages and Bremer Support values for most clades is poor.

The most significant result of this analysis is the recovery of all three named species of SML ichthyosaurs (*Janusaurus*, *Cryopterygius* and *Palvennia*) and PMO 222.667 in a single polytomy within Ophthalmosaurinae, which in turn is the sister group to another high latitude taxon, *Athabascasaurus*. Although relationships among the SML ichthyosaurs are not resolved, it does suggest the presence of a single clade of Boreal ophthalmosaurids that existed just prior to the Jurassic-Cretaceous boundary. From a biogeographic standpoint, this result is intriguing in that it mirrors interpretations regarding endemicity among Boreal ammonites and onychites (cephalopod hooklets) at this same time [65], [66]. Mutterlose et al. [67] proposed that the Greenland-Norwegian segment of the NE Atlantic was the deepest section of the seaway between the Boreal and Tethys Seas, bordered at both ends by shallower waters and islands. As such, the effect of sea-level fluctuations can significantly alter the palaeobiogeographic distribution of marine faunas, with periods of regression enhancing provincialism [66]. Low local sea-level at this time could explain the varying regional degree of the provinciality of the faunal realms [67]. The presence of endemic marine faunas in the Boreal realm at this time is also important, in that it provides potential insight into the timing and nature of connectivity between the water masses of the Tethyan and Boreal realms during the opening of the North Atlantic rift basin. Although these results provide an intriguing hypothesis regarding the paleobiogeography of ophthalmosaurids, more robust interpretations will ultimately depend on the results of ongoing phylogenetic work on the clade, particularly with respect to increasing taxon sampling and expanding the numbers of taxonomically informative characters.

## Conclusions

*Janusaurus lundi* gen. et sp. nov. is a new ophthalmosaurid ichthyosaur from the early Volgian of Spitsbergen (Svalbard). It possesses a unique combination of cranial and post-cranial features, compared to other ophthalmosaurids, including autapomorphic features of the pelvic and pectoral girdles. This new species adds to our knowledge about the diversity of ophthalmosaurid ichthyosaurs from the Jurassic-Cretaceous boundary. Including *Janusaurus lundi*, nine genera of ichthyosaurs are now known from the Tithonian/Volgian, four of which are found in high latitude regions. Significantly, three of these are from the SML, and other new taxa are currently under study. Phylogenetic analysis indicates that all the Svalbard ophthalmosaurids nest within Ophthalmosaurinae, and they form an unresolved clade of Boreal ichthyosaurs. All of the described ophthalmosaurid taxa from Spitsbergen to date are endemic and are not referable to taxa from lower latitudes. Based on the available information both plesiosaur and ichthyosaur diversity may parallel ammonite diversity, in which there are unique boreal taxa at this time [15]. This sparks the question whether there was a complete marine connection towards the Tethys Sea the entire NE Atlantic at this time interval, or simply that it was inaccessible. Our knowledge of the Svalbard ophthalmosaurids is far from complete, as many specimens have yet to be described. The marine amniote material from the Slottsmøya Member will continue to expand our knowledge of the diversity and distribution of marine amniotes during the latest Jurassic.

## Supporting Information

Figure S1 **Medial view of the skull of** ***Janusaurus lundi*** **(PMO 222.654).** The general interpretation is visible below. Scale = 5 cm.
(TIF)

Figure S2 **Other material from** ***Janusaurus lundi*** **(PMO 222.654).** A-D: Right humerus; A: dorsal view; B: ventral view; C: anterior view; D: posterior view, scale = 5 cm. E-J: Right femur; E: dorsal view; F: ventral view; G: proximal view; H: distal view; I: anterior view; J: posterior view, scale = 1 cm. K: plesiosaur tooth collected in the vicinity, scale = 1 cm. L: articular in medial view, scale = 1 cm. Abbreviation: dp, dorsal process; dpc, deltopectoral crest; ff, fibula facet; pef, preaxial accessory element fact; tf, tibia facet; Uf, ulna facet; vp, ventral process.
(TIF)

Table S1 Datamatrix for the phylogenetic analysis. Abbreviations: A, (01); B, (12).
(DOCX)

Text S1 **Description of characters used in the phylogenetic analysis.**
(DOCX)

Text S2 **Methods for the phylogeny and critical review of characters.**
(DOCX)

Video S1 **A complied photogrammetry video of the skull of** ***Janusaurus lundi*** **(PMO 222.654).**
(MP4)
