# Supplementary figures and images for: A New Upper Jurassic Ophthalmosaurid Ichthyosaur from the Slottsmøya Member, Agardhfjellet Formation of Central Spitsbergen
Source: PLoS One. 2014 Aug 1;9(8):e103152. doi: 10.1371/journal.pone.0103152 (PMC4118863; doi:10.1371/journal.pone.0103152)

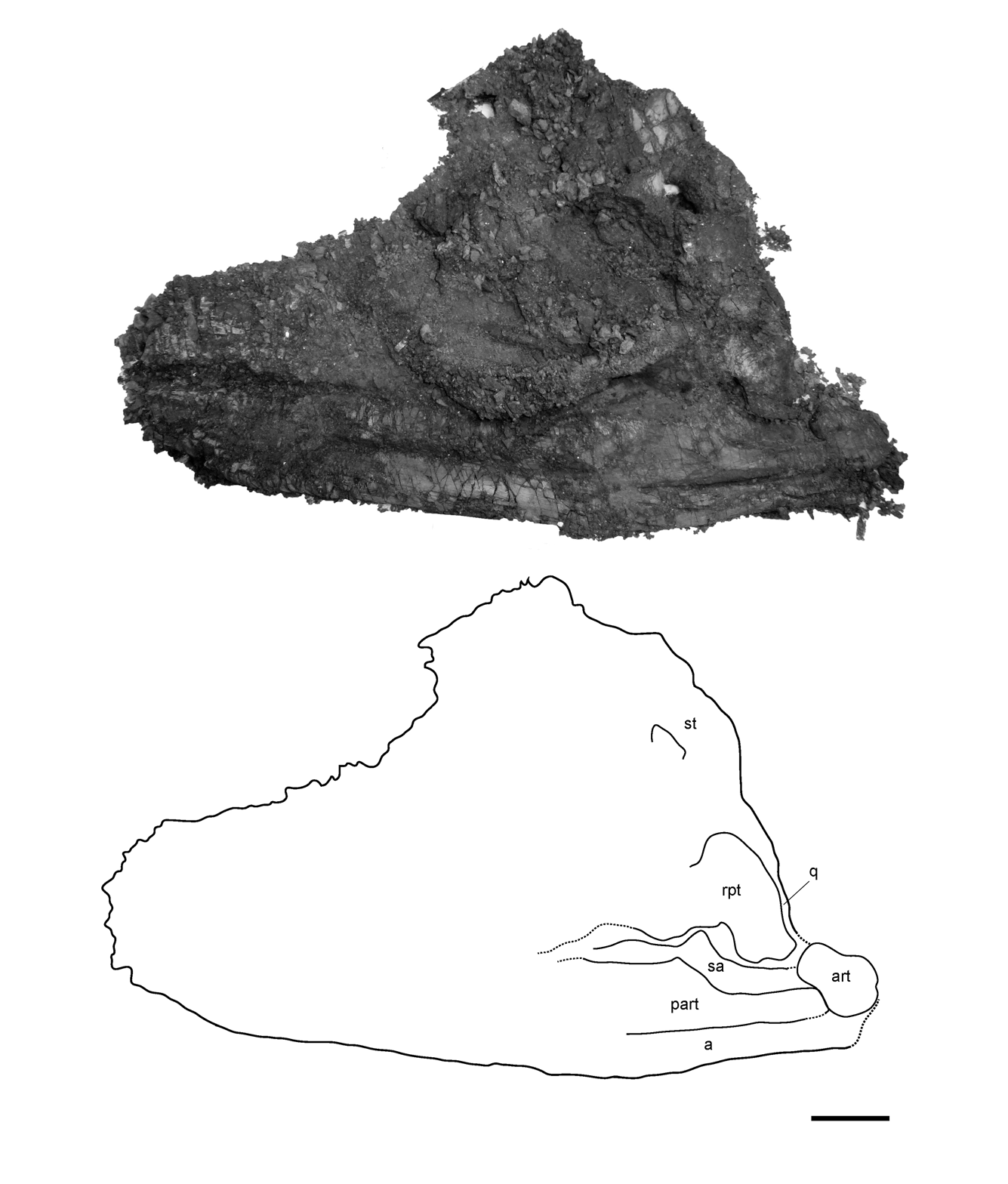

Supplement: Figure S1 — Medial view of the skull of Janusaurus lundi (PMO 222.654). The general interpretation is visible below. Scale = 5 cm. (TIF) [file pone.0103152.s001.tif]

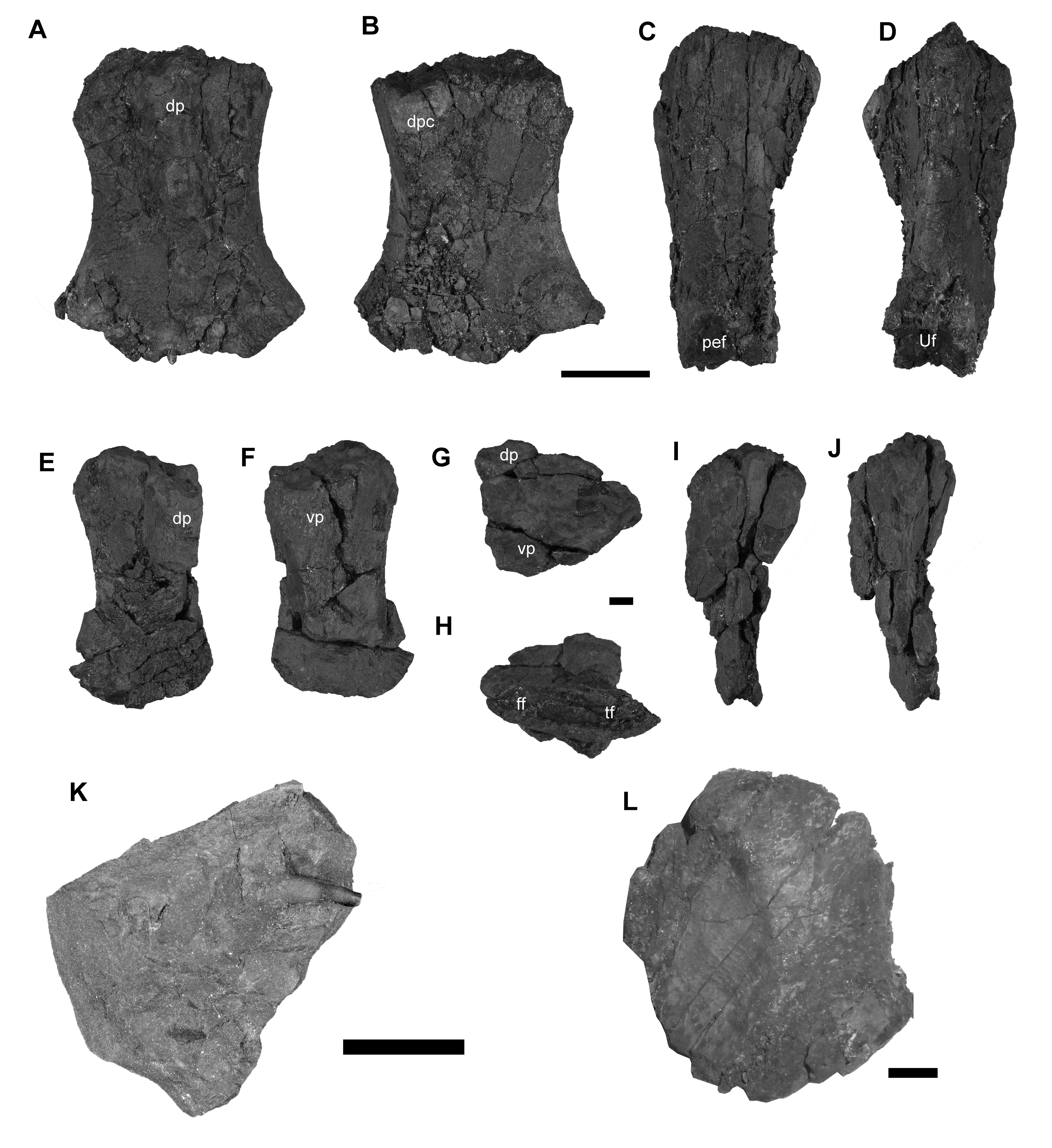

Supplement: Figure S2 — Other material from Janusaurus lundi (PMO 222.654). A-D: Right humerus; A: dorsal view; B: ventral view; C: anterior view; D: posterior view, scale = 5 cm. E-J: Right femur; E: dorsal view; F: ventral view; G: proximal view; H: distal view; I: anterior view; J: posterior view, scale = 1 cm. K: plesiosaur tooth collected in the vicinity, scale = 1 cm. L: articular in medial view, scale = 1 cm. Abbreviation: dp, dorsal process; dpc, deltopectoral crest; ff, fibula facet; pef, preaxial accessory element fact; tf, tibia facet; Uf, ulna facet; vp, ventral process. (TIF) [file pone.0103152.s002.tif]
