# Supplementary material for: A New Upper Jurassic Ophthalmosaurid Ichthyosaur from the Slottsmøya Member, Agardhfjellet Formation of Central Spitsbergen
Source: PLoS One. 2014 Aug 1;9(8):e103152. doi: 10.1371/journal.pone.0103152 (PMC4118863; doi:10.1371/journal.pone.0103152)
Supplement: Table S1 — Datamatrix for the phylogenetic analysis. Abbreviations: A, (01); B, (12). (DOCX) [file pone.0103152.s003.docx]

| **Table S1: Data matrix for the phylogenetic analysis.** Abbreviations: A, (01); B, (12). | | | | | | | |
| --- | --- | --- | --- | --- | --- | --- | --- |
| **Taxa** | **Characters** | | | | | | **Completeness** |
| *Temnodontosaurus sp.* | 0000000000 | 0000000000 | 0000000000 | 0000000000 | 0000000000 | 000000 | 100 % |
| *Ichthyosaurus communis* | 0000100A00 | 000011B100 | 0000000000 | A000010000 | A110101100 | 000010 | 100 % |
| *Stenopterygius quadriscissus* | 1001100000 | 0000010100 | 01010011?1 | 1100000000 | 10A0000110 | ??0000 | 94.6 % |
| *Ophthalmosaurus icenicus* | 0101111101 | 00100101A1 | A011011011 | 0101011101 | 1111110010 | 100010 | 100 % |
| *Brachypterygius extremus* | 011?0100?1 | 10??????12 | 11???1???? | ?101?10010 | 11111100?? | ?????? | 55.3 % |
| *Maiaspondylus lindoi* | ?11?1?01?1 | 0???????0? | ??????0??? | ???1?10010 | 1?1?0?10?? | ??10?? | 39.2 % |
| *Undorosaurus gorodischensis* | 111??????? | ??0??????? | ???????1?? | ?001?1110? | 11?1010?00 | 110010 | 46.4 % |
| *Arthropterygius chrisorum* | ?????????? | ????????02 | 11????11?1 | ??01001100 | ?1?1010??? | 1100?? | 42.8 % |
| *Mollesaurus periallus* | 11??1?0111 | ????001111 | 11010?0??? | ?????????? | ?????????? | ?????? | 28.6 % |
| *Acamptonectes densus* | 11???1???? | ??1?????11 | 11111100?1 | 0101A11101 | 1??1??0??? | ?????? | 50 % |
| *Caypullisaurus bonapartei* | ????00000? | 10??1?10?? | ?????10??? | 1111111000 | 11210110?? | 1?0?11 | 60.7 % |
| *Aegirosaurus leptospondylus* | 000?110111 | 00?10001?? | ?????1???? | ???1?10010 | 11111?1021 | ??0011 | 64.3 % |
| *Platypterygius australis* | 0110001001 | 1001012012 | 1101010111 | 0111111000 | 1121011020 | 110111 | 100 % |
| *Platypterygius hercynicus* | 011?100?01 | ??110??1?2 | ?1?10??1?1 | 0111111000 | 11210110?? | 11101? | 73.2 % |
| *Athabascasaurus bitumineus* | 10??0011?1 | 01100110?2 | ???1110?1? | ?????????? | ????????20 | 1????? | 42.8 % |
| *Malawania anachronus* | ?????????? | ?????????? | ?????????? | 00??110000 | ?0001?00?? | ?????? | 25 % |
| *Leninia stellans* | ?????10?01 | 0010?0A1?1 | ?0???1???? | ?????????? | ?????????? | ?????? | 25 % |
| *Sveltonectes insolitus* | 101?110111 | 0111?1??02 | 11?1?100?1 | 110111000? | ?111111021 | 110111 | 83.9 % |
| *Palvennia hoybergeti* | 11??00?11? | ??10112??2 | 11??11???? | ??????1??? | ???1?????? | ?????? | 32.1 % |
| *Cryopterygius kristiansenae* | 111?0001?0 | 001?1121?2 | ?????1???? | 0101010000 | 0111111000 | 11001? | 75 % |
| *Janusaurus lundi* | 11??010?10 | 011?1121?2 | 11??11???? | ??01001100 | 01?1110020 | 1100?? | 71.4 % |
| PMO 222.667 | 111??0???? | ????????12 | 11???????? | 0111001100 | 01?1110??? | ?????? | 41.1% |
