# Supplementary material for: A New Upper Jurassic Ophthalmosaurid Ichthyosaur from the Slottsmøya Member, Agardhfjellet Formation of Central Spitsbergen
Source: PLoS One. 2014 Aug 1;9(8):e103152. doi: 10.1371/journal.pone.0103152 (PMC4118863; doi:10.1371/journal.pone.0103152)
Supplement: Text S1 — Description of characters used in the phylogenetic analysis. (DOCX) [file pone.0103152.s004.docx]

**Text S1 Description of characters used in the phylogenetic analysis.** The characters are modified from those provided by Fischer *et al.* [23] and the references therein, *w*ith the exception of three new characters. New characters (11, 12, 16, 25, 52) are indicated with a “*”.

Skull

1. Crown striation: presence of deep longitudinal ridges (0); crown enamel subtly ridged or smooth (1).
2. Base of enamel layer: poorly defined, invisible (0); well defined, precise (1).
3. Root cross-section in adults: rounded (0); quadrangular (1).
4. Processus postpalatinis pterygoidei: absent (0); present (1).
5. Maxilla anterior process: extending anteriorly as far as nasal or further anteriorly (0) ; reduced (1).
6. Descending process of the nasal on the dorsal border of the nares: absent (0), present (1).
7. Processus narialis of the maxilla in lateral view: absent (0); present (1).
8. Processus supranarialis of the premaxilla: present (0); absent or reduced (1).
9. Processus narialis of the prefrontal: present (0); absent (1).
10. Anterior margin of the jugal: tapering, running between lacrimal and maxilla (0); broad and fan-like, covering large are of maxilla venterolaterally (1).
11. Anterior margin of the jugal: terminates prior to anterior end of lacrimal (0), reaches or surpasses anterior end of lacrimal (1). *
12. Posterior margin of the jugal: articulates with the postorbital and quadratojugal (0); excluded from the quadratojugal by the postorbital (1) *
13. Sagittal eminence: present (0); absent (1).
14. Processus temporalis of the frontal: absent (0); present (1).
15. Supratemporal-postorbital contact: absent (0); present (1).
16. Broad postfrontal-postorbital contact, absent (0), present (1).*
17. Squamosal shape: triangular (0); squared (1); squamosal absent (2).
18. Quadratojugal exposure: extensive (0): small, largely covered by squamosal or supratemporal and postorbital (1).
19. Basiopterygoid processes: short, giving basisphenoid a square outline in dorsal view (0); markedly expanded laterally, being wing-like, giving basisphenoid a marked pentagonal shape in dorsal view (1).
20. Extracondylar area of basioccipital: wide (0); reduced but still present ventrally and lateral (1); extremely reduced, being nonexistent at least ventrally (2).
21. Basioccipital peg: present (0); absent (1).
22. Ventral notch in the extracondylar area of the basioccipital: present (0); absent (1).
23. Shape of the paroccipital process of the ophisthotic: short and robust (0); elongated and slender (1).
24. Stapes proximal head: slender, much smaller than opisthotic proximal head (0); massive, as large or larger than ophistotic head (1).
25. Stapedial shaft in adults: thick (0), slender and gracile (1).*
26. Angular lateral exposure: much smaller than surangular exposure (0), extensive (1).

Axial skeleton

1. Posterior dorsal/anterior caudal centra: 3.5 times or less as high as long (0); four times or more as high as long (1).
2. Tail fin centra: strongly laterally compressed (0); as wide as high (1).
3. Neural spines of atlas-axis: completely overlapping, may be fused (0), never fused (1).
4. Chevrons in apical region: present (0); lost (1).

Appendicular skeleton

1. Glenoid contribution of the scapula: extensive, being at least as large as the coracoid facet (0); reduced, being markedly smaller than the coracoid facet (1).
2. Prominent acromion process of scapula: absent (0); present (1).
3. Anteromedial process of coracoid: Present (0); absent (1).

Forefin

1. Plate-like dorsal ridge on humerus: absent (0); present (1).
2. Protruding triangular deltopectoral crest on the humerus: absent (0); present (1).
3. Humerus distal and proximal ends in dorsal view: distal end wider than proximal end (0), nearly equal or proximal end slightly wider than distal end (1).
4. Humerus anterodistal facet for preaxial accessory element anterior to radius; absent (0); present (1).
5. Posteriorly deflected ulnar facet: absent (0); present (1).
6. Humerus/intermedium contact: absent (0); present (1).
7. Shape of the posterior surface of the ulna: rounded or straight and nearly as thick as the rest of the element (0); concave and edgy (1).
8. Manual pisiform: absent (0), present (1).
9. Notching of anterior facet of leading edge elements of forefin in adults: present (0); absent (1).
10. Posterior enlargement of forefin: number of postaxial accessory “complete” digits: none (0); one (1), two or more (2).
11. Preaxial accessory digits on forefin: absent (0); present (1).
12. Longipinnate or latipinnate forefine construction: one (0); two (1) digits directly supported by the intermedium.
13. Zeugo- to autopodial elements flattened and plate-like (0); strongly thickened (1).
14. Tightly packed rectangular phalanges: absent, phalanges are mostly rounded (0), present (1).
15. Digital bifurcation: Absent (0); frequently occurs in digit IV (1).

Pelvic girdle

1. Ischium-pubis fusion in adults: absent or present only proximally (0); present with an oburator foramen (1); present with no oburator foramen (2).
2. Ischium or ischiopubis shape: plate-like, flattened (0); rod-like (1).

Hindfin

1. Prominent, ridge-like dorsal and ventral process demarked from the head of the femur and extending up to the mid-shaft: absent (0); present (1).
2. Ventral process on femur: smaller than dorsal process (0), more prominent (1).*
3. Astragalus/femoral contact: absent (0); present (1).
4. Femur anterodistal facet for accessory zeugopodial element anterior to tibia: absent (0); present (1).
5. Tibia peripheral shaft in adults: notched (0); straight (1).
6. Postaxial accessory digit: absent (0); present (1).
