# Supplementary material for: A New Upper Jurassic Ophthalmosaurid Ichthyosaur from the Slottsmøya Member, Agardhfjellet Formation of Central Spitsbergen
Source: PLoS One. 2014 Aug 1;9(8):e103152. doi: 10.1371/journal.pone.0103152 (PMC4118863; doi:10.1371/journal.pone.0103152)
Supplement: Text S2 — Methods for the phylogeny and critical review of characters. (DOCX) [file pone.0103152.s005.docx]

**S2 Text: Methods for the phylogeny and critical review of characters.**

Methods and settings in TNT (Goloboff et al. 2010)

The phylogenetic data matrix was adapted from Fisher et al. 2012, with species added from *Fisher* et al. 2013a &b for *Leninia* and *Malawania*. *Undorosaurus gorodischensis* was coded from the literature provided by Efimov 1999.

Settings in TNT:

Maxtrees set to 10 000

Characters were not ordered or weighted.

The strict consensus tree was formed of 3 trees with 128 steps. The bootstrap was run with 1000 replicates, using the tree-bisection-reconnection (TBR) algorithm.

Critical review of characters

The characters used by Fisher *et al.* 2012 were critically reviewed and specimens for *Ophthalmosaurus icenicus* and *Brachypterygius extremus* were examined by AJR at the NHMUK, OUM and CAMSM. The scorings for the character states provided for these two taxa were found to be correct. Other taxa were reviewed from the literature. For *Arthropterygius* a number of characters were changed from the data provided by Fischer *et al.* 2012, based on the literature available for this genus (Maxwell 2010, Fernández & Maxwell 2012). The changes were made in the following characters, following Fischer *et al.* 2012 numbering of characters:

Character states changed for *Arthropterygius* from Fischer *et al.* 2012: 21 (1🡪?); 32 (1🡪0); 40 (?🡪1); 42 (?🡪1); (43 (?🡪0); 47 (0🡪1); 49 (?🡪0)

Character states changed for *Stenopterygius* from Fischer *et al.* 2012: 19 (0🡪1), as absent in most specimens (Maxwell et al. 2012)

Character states changed for *Caypullisaurus* from Fischer *et al.* 2012: 45 (2🡪?); 46 (1🡪?). This change is due to the insecurity around which element the pelvic element present in the holotype of *Caypullisaurus* represents.

Character states changed for *Platypterygius hercynicus* from Fischer *et al.* 2012: 14 (A🡪?). This has been changed to missing data, as no squamosal is actually preserved and the presence of this element is only inferred.

The character states for *Cryopterygius kristiansenae*, *Palvennia hoybergeti*, *Janusaurus lundi* and PMO 222.667 were scored from personal observation by AJR and JHH on the holotype specimens.

The data on completeness of the characters for the different taxa is reflected in Table S1. The majority of the taxa have enough data to score for over 50 % of the characters. Some taxa, which are described from fragmentary and incomplete material, have less than 30 % of the data scored. This is reflected in the low bootstrap values. More parsimony-informative characters and complete material are necessary to understand the phylogenetic relationships within the clade.
